# Molecular excitons in arylazopyrazole aggregates: a quantum chemical study

**DOI:** 10.1038/s41598-026-53659-1

**Published:** 2026-06-10

**Authors:** Anna Zehle, Christopher Penschke, Evgenii Titov

**Affiliations:** Institute of Chemistry, Theoretical Chemistry, University of Potsdam, Karl-Liebknecht-Straße 24-25, 14476 Potsdam, Germany

**Keywords:** Chemistry, Materials science, Optics and photonics, Physics

## Abstract

Aggregation of molecular photoswitches may affect their functionality. Fundamentally, interaction of monomers in the aggregated state results in formation of exciton states, which, in turn, govern energy and charge transfer processes in the materials made of the photoswitches. In this work, we study the exciton states of aggregates of arylazopyrazole — the photoswitch which gained popularity in last decade as an alternative to azobenzene — using quantum chemical calculations. We perform cluster excited-state calculations for aggregates including up to 32 arylazopyrazole monomers as well as periodic calculations for the crystal structure. We obtain and analyze the composition of the exciton states, exciton splittings, and monomer-to-aggregate spectral shifts, thus providing quantitative insight into the electronic states and absorption spectra of realistic arylazopyrazole aggregates.

## Introduction

Molecular switches are a fascinating class of dynamic compounds capable of undergoing reversible transformations between distinct states (isomers) upon exposure to external stimuli, such as heat or light^1–3^. These switches are fundamental components in a wide range of emerging technologies, including responsive materials, molecular machines, and smart energy systems^4–6^.

Of special interest are systems composed of many photoswitchable molecules which are located close to each other. Examples of such systems are molecular crystals^7,8^, self-assembled monolayers (SAMs)^9^, micelles^10^, metal-organic frameworks (MOFs) decorated with photoswitches^11^, polymers^12^, surfactant–DNA complexes^13,14^, and various supramolecular architectures^15,16^. The close proximity (where closeness is loosely defined by the strength of intermolecular interaction) enables formation of collective electronic states known as molecular excitons^17,18^.

Most commonly, these systems are based on azobenzene, the *trans* $\leftrightarrow$ *cis* photoswitch widely studied and used in variety of applications^19,20^. The formation of exciton states in azobenzene aggregates was explored in recent years using computational chemistry. The exciton states of azobenzene dimers were investigated with linear response time-dependent density functional theory (TD-DFT) and post-Hartree–Fock wavefunction-based methods (CC2, ADC(2))^21,22^ as well as semiempirical quantum chemical methods^23,24^. Also, advanced exciton models were devised and applied to the dimers^25,26^. Furthermore, the excited states of larger aggregates (up to 18 molecules) mimicking SAMs were studied with global hybrid and long-range corrected TD-DFT^27,28^. Apart from the non-covalent aggregates, covalently linked multiazobenzenes were also studied^29–33^. Moreover, periodic calculations were performed for azobenzene SAMs and MOFs in the framework of the Bethe–Salpeter equation (BSE) / Green’s function many-body perturbation theory (*GW*)^34–36^. Experimentally, spectral changes in UV/vis spectra were observed in the aggregated state and explained by the exciton formation^10,14,37–39^.

The world of photoswitches expands, however, beyond azobenzenes. One particular class of similar but different photoswitches are arylazopyrazoles, in which one phenyl ring is replaced with a pyrazole ring. The advantages of arylazopyrazoles are photostationary states with $\ge$ 90% of either *trans* or *cis* isomer (depending on the irradiation wavelength) and long thermal half-lives of the *cis* isomer (up to several years)^40^. Self-assembly of these photoswitches has been demonstrated at the air–water interface^41^ as well as in micelles of photosurfactants^42^. Also arylazopyrazole-containing SAMs on surfaces were recently realized and studied^43,44^. Moreover, single crystal structures were reported in several works^41,45,46^. However, little is known about molecular excitons of aggregated arylazopyrazoles.

In this work, we want to fill this knowledge gap by studying the molecular excitons of arylazopyrazole aggregates using quantum chemical calculations. In particular, we consider the aggregate geometries extracted from the experimentally determined crystal structure reported by Nagai et al.^41^ We analyze effects of aggregation direction and dimensionality of aggregates (1D, 2D, or 3D) on absorption spectra as well as on exciton composition and localization. We also report periodic TD-DFT calculations on the molecular crystal structure.

## Models and methods

### Models

**Fig. 1:**
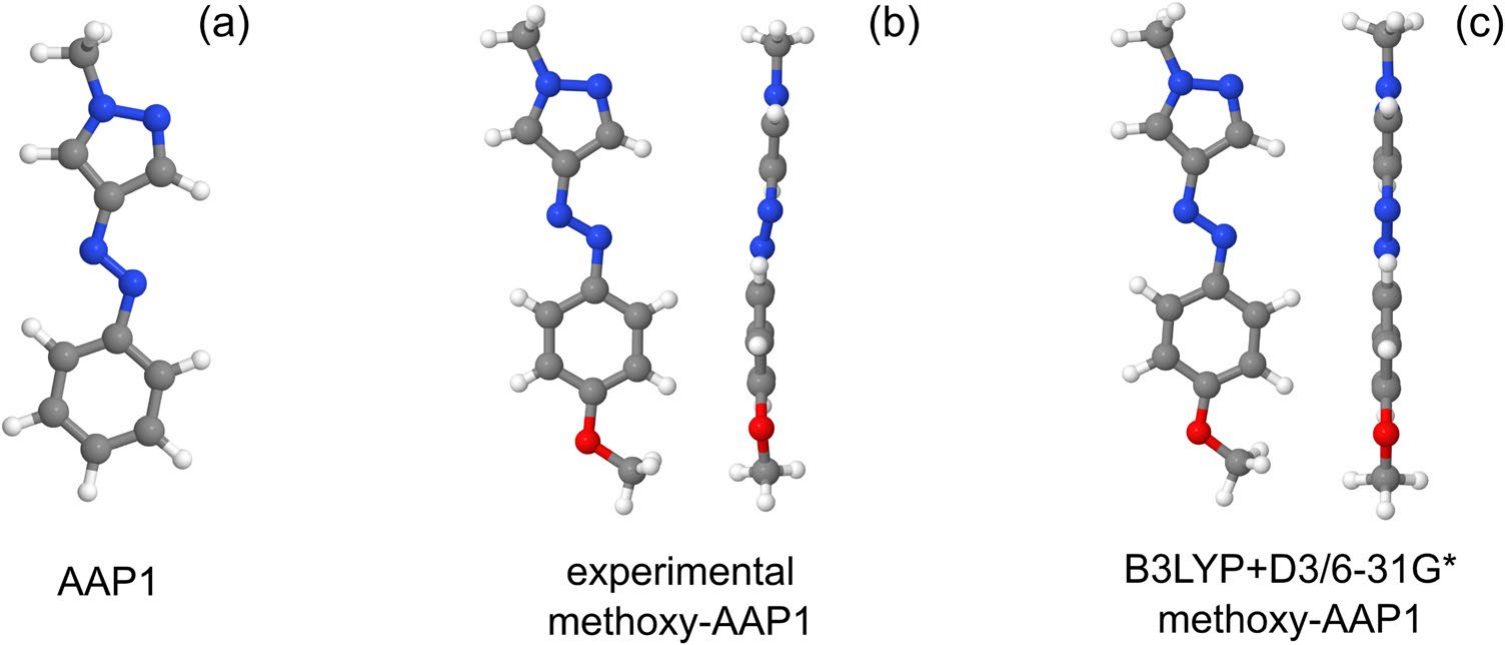
Monomers. Arylazopyrazoles (AAPs) investigated in this work. Panel (**a**) depicts the parent arylazopyrazole AAP1 optimized using B3LYP+D3(BJ)/6-31G*. In panels (**b**) and (**c**), its derivative methoxy-AAP1 is shown (in two different views for each panel). The structure of methoxy-AAP1 in (**b**) corresponds to the geometry determined by Nagai et al.^41^ using X-ray scattering. In (**c**), the geometry optimized at the B3LYP+D3(BJ)/6-31G* level of theory is shown.

The monomers of parent arylazopyrazole 4-[(1E)-2-phenyldiazenyl]-1-methyl-1H-pyrazole (AAP1) and its derivative 4-[(1E)-2-(4-methoxyphenyl)diazenyl]-1-methyl-1H-pyrazole (methoxy-AAP1) studied in this work are shown in Fig. 1. The geometries were optimized using density functional theory (DFT) (see section “Methods” for details). For methoxy-AAP1, we also considered the experimental geometry determined using X-ray scattering by Nagai et al.^41^ As can be seen in Fig. 1, the experimental geometry is slightly non-planar in contrast to the DFT-optimized geometry.

**Fig. 2:**
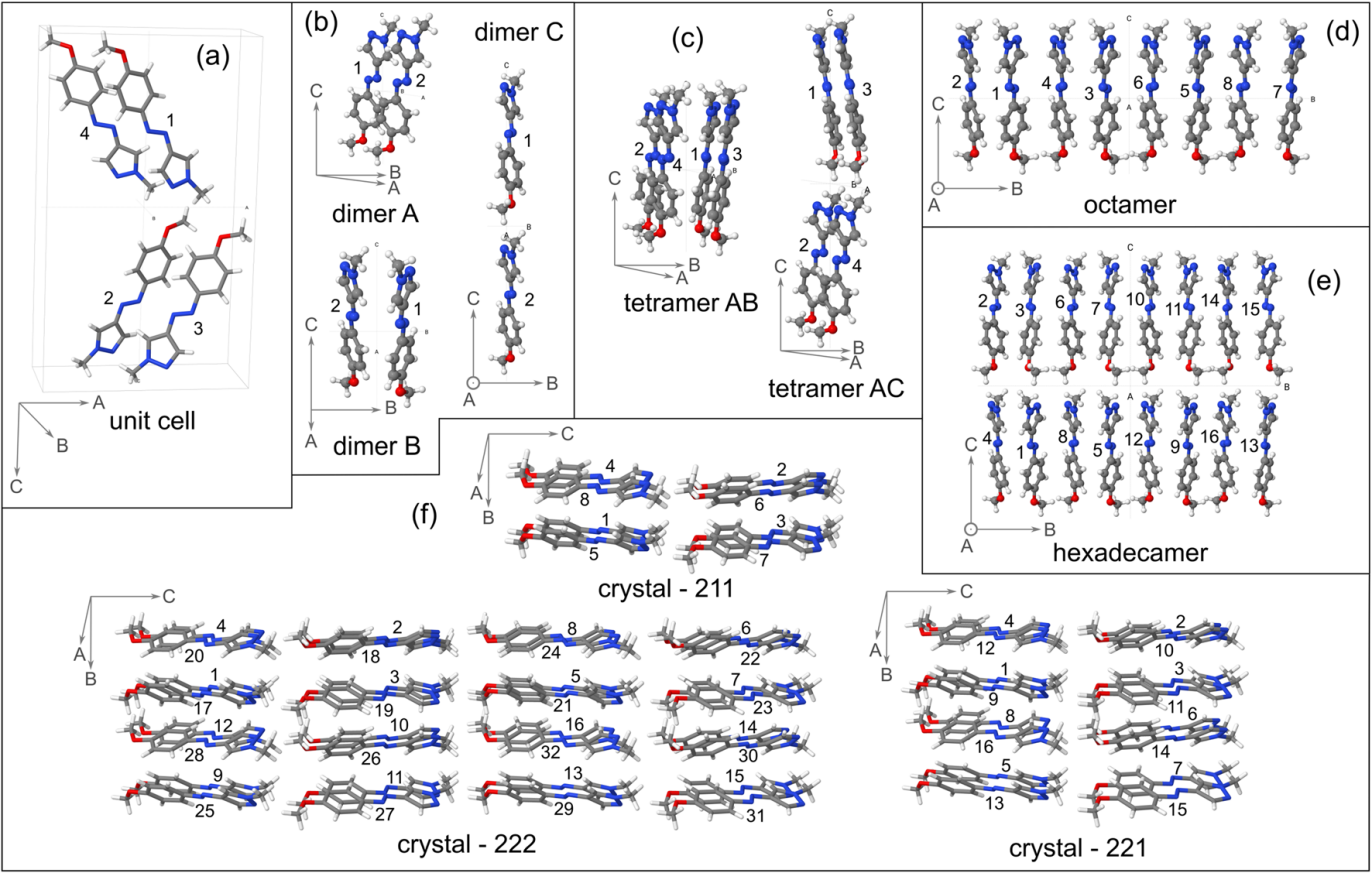
Aggregates. Model aggregates studied in this work. The aggregate geometries are extracted from experimentally determined crystal structure reported by Nagai et al.^41^ (**a**) Unit cell (consisting of four molecules, $N=4$). (**b**) Dimers A, B, and C ($N=2$). (**c**) Tetramers AB and AC ($N=4$). (**d**) Linear octamer ($N=8$). (**e**) 2D hexadecamer ($N=16$). (**f**) 3D structures crystal-211 ($N=8$), crystal-221 ($N=16$), and crystal-222 ($N=32$). Axes A, B, and C are shown for each aggregate. Monomer numbering is provided.

The experimental crystal structure of methoxy-AAP1^41^ was further used to extract various aggregate geometries consisting of *N* monomers, $N=2,4,8,16,32$ (Fig. 2). The unit cell of the crystal includes four molecules (Fig. 2a). First, three nearest-neighbour dimers ($N=2$), dimer A, dimer B, and dimer C, selected along the A, B, and C axes, respectively, were considered (Fig. 2b). More views of the dimers with key structural parameters are shown in Fig. S24. Moreover, the geometries of all considered aggregates are provided in the XYZ format at the link in the “Data availability﻿” section. Second, tetramers AB, AC (Fig. 2c), and “unit cell” (which is the unit cell of the crystal, and extends in directions BC, Fig. 2a) were studied. Then, a linear (1D) octamer ($N=8$) in the B direction (Fig. 2d) and 2D hexadecamer ($N=16$) in BC plane (Fig. 2e) were examined. Finally, 3D structures were investigated, namely octamer “crystal-211” ($N=8$), hexadecamer “crystal-221” ($N=16$), and dotriacontamer “crystal-222” ($N=32$), see Fig. 2f.

The crystal model (bulk) used in the periodic calculations (see section “Methods” below) was built based on the experimentally determined unit cell containing 4 methoxy-AAP1 molecules (Fig. 2a).

### Methods

For geometry optimization and frequency calculations of the monomers, the B3LYP functional^47,48^ with dispersion correction D3(BJ)^49^ was used in combination with two different basis sets: 6-31G*^50,51^ and def2-TZVP^52^. Excited-state calculations were carried out using linear response time-dependent density functional theory (TD-DFT)^53^ with the $\omega$B97X-D long-range corrected hybrid functional^54^, as the long-range correction is known to cure the charge-transfer problem in TD-DFT^55,56^. This problem is expected to occur for the molecular aggregates studied here, and the $\omega$B97X-D functional was chosen as a “workhorse” method based on our experience with azobenzene aggregates^28^. Mostly the moderate def2-SV(P) basis set^52^ was used considering the large size of the aggregates (up to 32 molecules). For comparison with periodic calculations (see below) also the PBE functional^57^ was used. Monomer spectra were also computed with B3LYP, CAM-B3LYP^58^ and configuration interaction singles (CIS) combined with the 6-31G*, def2-SV(P), and def2-TZVP basis sets. In addition, the wavefunction-based ADC(2) method^59^ and its spin-scaled versions^60,61^ SCS-ADC(2) and SOS-ADC(2) were used for the monomer and dimers of methoxy-AAP1. The cc-pVDZ basis set^62^ and the corresponding auxiliary basis set^63^ were used in these calculations. The calculations with additional methods for the monomers and dimers were carried out to quantitatively assess the performance of $\omega$B97X-D. We also note that the post-Hartree–Fock wavefunction-based ADC(2) method is too costly for the large aggregates. In the TD-$\omega$B97X-D calculations, 20 excited states were requested for the monomer and usually 50 excited states for the aggregates (20 for octamer, 70 for crystal-222). In the ADC(2) calculations, 10 excited states were requested (for the monomer and the dimers). In the TD-PBE calculations, 300 excited states were requested for crystal-211. All cluster (TD-)DFT calculations were performed using Gaussian 16 (Revision C.01)^64^, the ADC(2) calculations were carried out with TURBOMOLE V7.0^65,66^. In the Gaussian calculations, the IOP(9/40=4) and IOP(3/33=1) keywords were used to print more CI-type expansion coefficients and the atomic orbital overlap matrix (see below), respectively.

Absorption spectra were calculated from excitation energies and oscillator strengths as

1 $$\begin{aligned} I(E)= \sum \limits _{i} f_{i}\exp \left( -\dfrac{1}{2\sigma ^2}\left( E-E_{i}\right) ^2\right) \quad . \end{aligned}$$

Here, *I* is intensity, *E* is excitation energy, $E_{i}$ and $f_{i}$ are the calculated excitation energy and oscillator strength, respectively, for the $S_0 \rightarrow S_i$ transition, and $\sigma$ is a broadening parameter ($\sigma =0.1$ eV in this work).

Exciton (de)localization and charge transfer contributions were analyzed using the transition density matrix analysis^67,68^. Specifically, the “fraction of transition density matrix” (FTDM) matrix was calculated for a given transition as^22^

2 $$\begin{aligned} F_{XY} = \dfrac{\sum \limits _{\mu \in X} \sum \limits _{\nu \in Y}\left( \mathbf{S}^{1/2}\mathbf{P}^{\mathrm {[AO]}}\mathbf{S}^{1/2}\right) _{\mu \nu }^2 }{\sum \limits _{\mu \in \textrm{aggregate}} \sum \limits _{\nu \in \textrm{aggregate}}\left( \mathbf{S}^{1/2}\mathbf{P}^{\mathrm {[AO]}}\mathbf{S}^{1/2}\right) _{\mu \nu }^2} \quad . \end{aligned}$$

Here, $\mathbf{P}^{\mathrm {[AO]}}$ is the TDM in atomic orbital (AO) basis (computed with Multiwfn 3.8^69,70^) and $\mathbf{S}$ is the AO overlap matrix. Diagonal elements $F_{XX}$ quantify contributions of local excitations (LE) and off-diagonal elements $F_{XY} (\ X\ne Y$) charge transfer (CT) excitations (*X*, *Y* denote monomers of an aggregate; the used monomer numbering is provided in Fig. 2). In what follows, we will express the FTDM elements in %, *i.e.,* as $F_{XY}\!\times\!100\!\%$. In addition, the natural transition orbital (NTO) analysis^71^ was conducted for selected systems.

Periodic DFT and TD-DFT calculations were performed using VASP^72,73^, version 6.5.0, employing projector-augmented wave (PAW)^74,75^ pseudopotentials. The PBE functional^57^ was used with a kinetic energy cutoff of 400 eV for the plane-wave basis set. The SCF convergence condition was set to 10$^{-6}$ eV. A 8$\times$7$\times$2 k-point grid was used for the crystal, which has unit cell dimensions of 6.109 Å $\times$ 7.0129 Å $\times$ 25.0189 Å. Monomer calculations were performed using only the $\Gamma$ point with a supercell of dimensions 20 Å $\times$ 17 Å $\times$ 25 Å, such that the minimum distance between atoms of periodic replica is at least 14 Å. In addition, we performed calculations using the B3LYP functional^47,48^ with the same settings as in the PBE calculations, except for the k-point grid in the crystal calculations, which was reduced to 4$\times$3$\times$1. The calculations were performed on the experimental crystal geometry^41^. For test purposes, the crystal structure was also reoptimized at the PBE+D3(BJ) level.

The imaginary part of the frequency-dependent dielectric function, corresponding to an absorption spectrum, was calculated using three different methods. The first is the Green–Kubo formula (GK; LOPTICS=.True. in VASP)^76^. The other two involve solving the Casida equation, either within the Tamm–Dancoff approximation (TDA) or using a full approach (TD-DFT)^77^. The GK approach uses all available occupied and unoccupied bands; adding more unoccupied states by increasing the total number of bands may affect the obtained spectrum. We used 128 bands (41 occupied and 87 unoccupied) for the monomer and 512 bands (164 occupied and 348 unoccupied) for the crystal. Test calculations using the GK approach showed that the changes within the energy range we consider in this work (up to around 6 eV) are negligible when the number of bands is increased from 128 to 1024 for the monomer or from 512 to 2048 for the crystal (see Fig. S18). TDA and TD-DFT calculations are much more demanding in terms of computational resources (particularly memory), so the number of bands that are included in crystal calculations has to be limited. We used 16 occupied and 16 unoccupied bands for the crystal, and for consistency we compare the results to monomer calculations with 4 occupied and 4 unoccupied bands (*i.e.*, we used the same amount of bands per monomer in both cases).

## Results and discussion

### Monomers

**Fig. 3:**
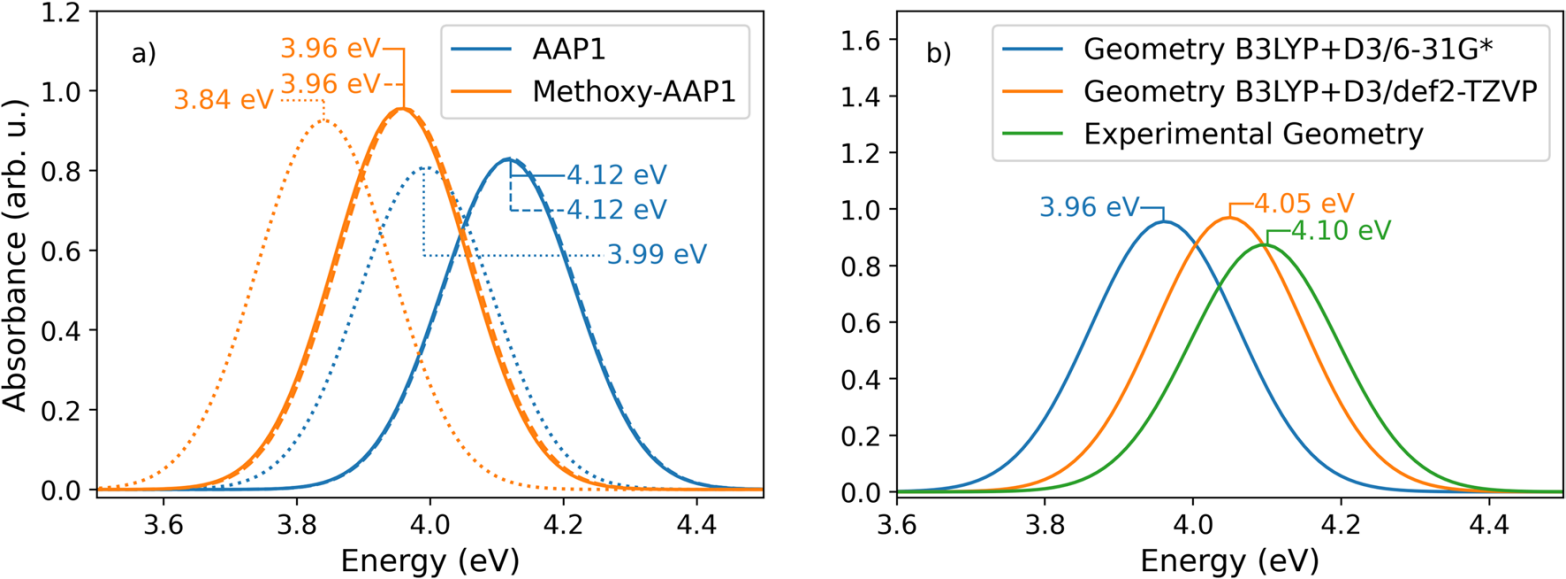
Spectra of monomers. (**a**) The $\pi \pi ^*$ absorption bands of methoxy-AAP1 (orange) and AAP1 (blue). The geometries of both molecules were optimized at the B3LYP+D3(BJ)/6-31G* level of theory. The dashed curves were calculated using TD-$\omega$B97X-D/def2-SV(P), the solid curves with TD-$\omega$B97X-D/6-31G*, and the dotted curves with TD-$\omega$B97X-D/def2-TZVP. (**b**) The $\pi \pi ^*$ absorption band of methoxy-AAP1 for different geometries (see legend) calculated using the TD-$\omega$B97X-D/def2-SV(P). The peak positions are provided.

The vertical, broadened absorption spectra of the two monomers (parent AAP1 and methoxy-substituted methoxy-AAP1) calculated using TD-$\omega$B97X-D in combination with three basis sets (6-31G*, def2-SV(P), and def2-TZVP) at the B3LYP+D3(BJ)/6-31G* optimized geometries are shown in Fig. 3a.

The methoxy substitution leads to a red shift of the absorption band in comparison to the parent compound. Specifically, the peak positions are 4.12 eV for AAP1 and 3.96 eV for methoxy-AAP1 when using the double-zeta 6-31G* or def2-SV(P) basis sets, and 3.99 eV for AAP1 and 3.84 eV for methoxy-AAP1 when using the triple-zeta def2-TZVP basis set. [We note that the experimental peak position for methoxy-AAP1 in aqueous solution is 3.57 eV (347 nm)^41^. The solvent effect, however, is not considered in our calculations, as our primary focus is on the spectral changes induced by aggregation.] The red shift “AAP1 minus methoxy-AAP1” is thus 0.16 eV with the double-zeta basis sets and 0.15 eV with the triple-zeta basis set. It appears to be almost unaffected by enlarging the basis. However, going from double-zeta to triple-zeta red-shifts the peak positions of each monomer by 0.12–0.13 eV (Fig. 3a). We also note that the spectra obtained with the 6-31G* and def2-SV(P) basis sets are virtually identical to each other (for the same molecule). Furthermore, the methoxy substitution also slightly increases absorbance in comparison to that of parent AAP1.

Next, we studied how geometry of the methoxy-AAP1 affects its absorption band. Fig. 3b shows TD-$\omega$B97X-D/def2-SV(P) spectra calculated at three different geometries: (i) optimized with B3LYP+D3(BJ)/6-31G*, (ii) optimized with B3LYP+D3(BJ)/def2-TZVP, and (iii) the experimental geometry. The obtained peak positions are 3.96 eV, 4.05 eV, and 4.10 eV for (i), (ii), and (iii), respectively. Thus, the use of the experimental geometry without reoptimization leads to a blue shift of 0.14 eV and 0.05 eV in comparison to the peak positions obtained at the geometries optimized with the 6-31G* and def2-TZVP basis sets, respectively. The absorbance at maximum is slightly lower when using the experimental geometry (Fig. 3b). We note that the basis set dependence observed in Fig. 3a also holds for the experimental geometry of methoxy-AAP1 (Fig. S1).

**Fig. 4:**
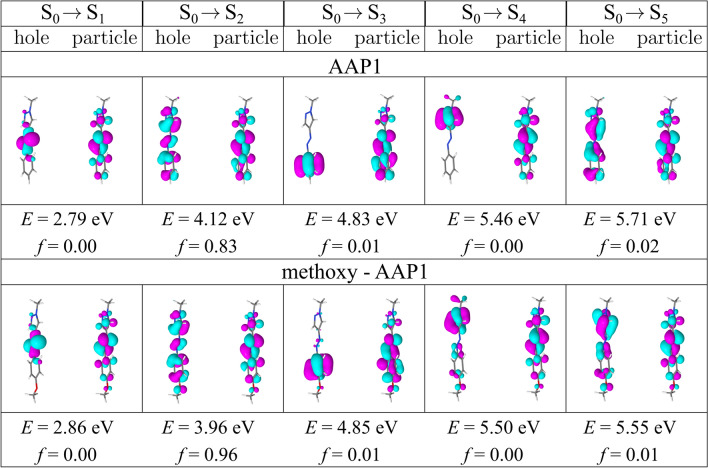
Monomer NTOs. The natural transition orbitals (NTOs) of AAP1 and methoxy-AAP1 for electronic transitions from $S_0 \rightarrow S_1$ to $S_0 \rightarrow S_5$. The geometries of both molecules were optimized at the B3LYP+D3(BJ)/6-31G* level of theory. Excited-state calculations were performed using TD-$\omega$B97X-D/def2-SV(P). Excitation energies (*E*) and oscillator strengths (*f*) are provided.

The band shown in Fig. 3 corresponds to the $S_0 \rightarrow S_2$ transition, which is a $\pi \pi ^*$ transition as revealed by NTOs presented in Fig. 4. The hole and particle $S_0 \rightarrow S_2$ NTOs of methoxy-AAP1 show the involvement of the oxygen atom, which correlates with the observed red shift upon methoxylation. The $S_0 \rightarrow S_1$ transition is a dark $n\pi ^*$ transition located at $\sim$2.8 eV as common for azo-switches. The higher-lying states ($S_3,S_4,S_5$) are $\pi \pi ^*$ states (see Fig. 4). We note that oxygen contributes to hole and particle NTOs for these states, in the case of methoxy-AAP1.

The absorption spectra obtained with additional TD-DFT methods (B3LYP and CAM-B3LYP) and CIS for AAP1 and methoxy-AAP1 are presented in Fig. S2. In all cases, B3LYP leads to a red shift, while CIS calculations produce a pronounced blue shift (see also Table S1). CAM-B3LYP and $\omega$B97X-D lead nearly to identical results, with only minor variations depending on the chosen basis set (Fig. S2 and Table S1).

Further, we also performed ADC(2) as well as SCS-ADC(2) and SOS-ADC(2) calculations for the methoxy-AAP1 monomer, see Fig. S3. The $\pi \pi ^*$ peak positions are 4.11, 4.30, and 4.38 eV for ADC(2), SCS-ADC(2), and SOS-ADC(2), respectively, all with the cc-pVDZ basis set and at the experimental geometry. The ADC(2) value of 4.11 eV is very close to the $\omega$B97X-D value of 4.10 eV (see also Table S5 for “$\omega$B97XD minus ADC(2)” excitation energy differences for the monomer and the dimers). The spin-scaling blue-shifts the absorption band (the blue shift is larger for SOS than for SCS). At the B3LYP+D3(BJ)/6-31G* optimized geometry, the peak positions are 3.98, 4.15, and 4.23 eV for ADC(2), SCS-ADC(2), and SOS-ADC(2), respectively. Again, the ADC(2) value (3.98 eV) is close to the $\omega$B97X-D result (3.96 eV, see Fig. 3b).

### Dimers

**Fig. 5:**
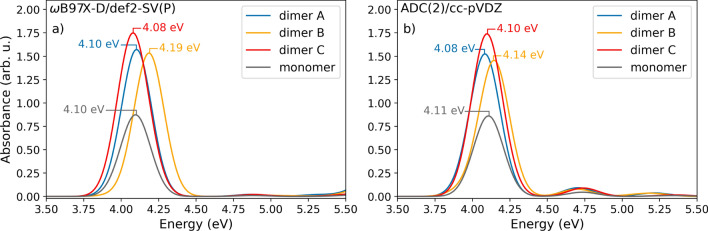
Spectra of dimers. The $\pi \pi ^*$ absorption bands of the dimers and the monomer. (**a**) TD-$\omega$B97X-D/def2-SV(P) spectra. (**b**) ADC(2)/cc-pVDZ spectra. The peak positions are provided.

We studied three dimers (dimer A, dimer B, and dimer C) extracted from the crystal structure, see Fig. 2b. Their absorption spectra calculated with TD-$\omega$B97X-D/def2-SV(P) and ADC(2)/cc-pVDZ are shown in Fig. 5.

At the TD-$\omega$B97X-D/def2-SVP level (Fig. 5a), the peak positions are 4.10 eV, 4.19 eV, and 4.08 eV for dimers A, B, and C, respectively. For the monomer (experimental geometry), the $\pi \pi ^*$ absorption peak is at 4.10 eV. Thus, for dimer A, virtually no spectral shift is observed with respect to the monomer [the “exact” values in the Gaussian output are 4.0960 eV for the monomer and 4.1038 eV for dimer A]. We note that in the case of dimer A, transition dipole moments of the monomers (for the monomeric $S_0 \rightarrow S_2$ transition) build an angle of 58.6$^\circ$ with respect to the intermolecular axis (Fig. S4). The Kasha exciton theory predicts zero exciton coupling for the case of “magic angle” of 54.7$^\circ$ ($\arccos \frac{1}{\sqrt{3}}$) and no spectral shift is expected at a slightly larger angle (assuming that the van der Waals displacement is small)^18^. In general, however, the zero shift occurs if the magnitude of exciton coupling equals the magnitude of the van der Waals displacement^18,21^. As will be discussed below, this situation occurs here for dimer A, *i.e.*, both the exciton coupling and the van der Waals displacement are non-zero (see also Fig. S22 showing the exciton splitting). Dimer B instead exhibits a blue shift (hypsochromic shift) of 0.09 eV with respect to the monomer. This is characteristic of the so-called H-aggregates^78^. In contrast, dimer C demonstrates a slight red shift (bathochromic shift) of 0.02 eV, characteristic of the so-called J-aggregates (with weak exciton coupling)^78^. At the ADC(2)/cc-pVDZ level (Fig. 5b), a qualitatively similar picture is observed with exception of slight red shift for dimer A. Quantitatively, the peak positions are 4.08 eV, 4.14 eV, and 4.10 eV for dimers A, B, and C, respectively, and 4.11 eV for the monomer. This results in a red shift of 0.03 eV for dimer A, a blue shift of 0.03 eV for dimer B, and a red shift of 0.01 eV for dimer C. We emphasize that spectral shifts are always calculated within the framework of a single method, and the small shifts are meaningful.

The $\pi \pi ^*$ exciton splittings and monomer-to-dimer spectral shifts (“dimer − monomer”) are summarized in Table  1. The exciton splittings were calculated as differences of excitation energies for states $S_3$ and $S_4$ of a dimer (*d*), namely $E^{(d)}_{S_4} - E^{(d)}_{S_3}$. The spectral shifts which we term “state shifts” were obtained as differences between excitation energies of the brightest states (*b*) of the monomer (*m*) and the dimer (*d*), $E^{(d)}_b-E^{(m)}_b$. Here, $E^{(m)}_b$ is $E^{(m)}_{S_2}$, and $E^{(d)}_b$ is $E^{(d)}_{S_4}$ for dimers A and B, and $E^{(d)}_{S_3}$ for dimer C (see below). The excitation energies and oscillator strengths for the monomer and the dimers are summarized in Table 2.

**Table 1:** Dimer exciton splittings and spectral shifts.

Dimer	$\omega$B97X-D/def2-SV(P)			ADC(2)/cc-pVDZ		
	Exciton splitting (eV)	Peak shift (eV)	State shift (eV)	Exciton splitting (eV)	Peak shift (eV)	State shift (eV)
A	0.12	0.00	0.01	0.11	−0.03	−0.02
B	0.24	0.09	0.09	0.20	0.03	0.04
C	0.07	−0.02	−0.03	0.06	−0.01	−0.02

Exciton splittings and monomer-to-dimer spectral shifts for methoxy-AAP1 dimers A, B, and C calculated with TD-$\omega$B97X-D/def2-SV(P) and ADC(2)/cc-pVDZ. The “peak shifts” were determined from the maxima of the spectra, whereas the “state shifts” were obtained using excitation energies of the brightest states.

**Table 2:** Excitation energies and oscillator strengths.

State	Monomer		Dimer A		Dimer B		Dimer C	
	*E* (eV)	*f*	*E* (eV)	*f*	*E* (eV)	*f*	*E* (eV)	*f*
	$\omega$B97X-D/def2-SV(P)							
$S_1$	2.91	0.00	2.91	0.00	2.89	0.00	2.90	0.00
$S_2$	**4.10**	**0.87**	2.93	0.00	2.90	0.00	2.91	0.00
$S_3$	4.88	0.01	3.99	0.03	3.94	0.01	**4.06**	**1.37**
$S_4$	5.46	0.00	**4.10**	**1.56**	**4.19**	**1.53**	4.14	0.46
$S_5$	5.57	0.01	4.83	0.01	4.70	0.00	4.88	0.01
	ADC(2)/cc-pVDZ							
$S_1$	3.24	0.00	3.22	0.00	3.22	0.00	3.23	0.00
$S_2$	**4.11**	**0.86**	3.25	0.00	3.22	0.00	3.24	0.00
$S_3$	4.73	0.05	3.98	0.09	3.95	0.07	**4.08**	**1.35**
$S_4$	5.26	0.01	**4.09**	**1.47**	**4.15**	**1.45**	4.14	0.46
$S_5$	5.40	0.01	4.70	0.03	4.67	0.00	4.73	0.05

Excitation energies (*E*) and oscillator strengths (*f*) for the lowest five excited states of the methoxy-AAP1 monomer and its dimers, calculated using TD-$\omega$B97X-D/def2-SV(P) and ADC(2)/cc-pVDZ. The brightest transitions are highlighted in bold.

The exciton splittings decrease in the order dimer B > dimer A > dimer C (Table 1). Interestingly, the splitting drops by a factor of $\sim$2 when going from B to A, and again by the same factor when going from A to C. The same trend is observed with both $\omega$B97X-D and ADC(2). The spectral “state shifts” are similar to those discussed above, obtained from peak positions of the broadened spectra (“peak shifts”). However, we note that non-zero oscillator strength for the less intense state of the $\pi \pi ^*$ exciton-split pair (see Table 2) leads to a slight quantitative difference when computing the shifts from the peak positions. As already noted above based on the broadened spectra, both methods $\omega$B97X-D and ADC(2) qualitatively agree on the sign of the shift for dimers B and C, but not for dimer A — TD-DFT predicts a small blue shift (0.01 eV), whereas ADC(2) yields a small red shift ($-0.02$ eV). Moreover, for dimer A, the small spectral shift ($\sim$0.01 eV) comes with much larger exciton splitting ($\sim$0.1 eV) (Table 1); therefore, the almost zero spectral shift is a result of counteracting van der Waals displacement and exciton coupling of similar magnitudes ($\sim$0.05 eV).

From Table 2, we see that the brightest state is $S_4$ (the upper state of the exciton-split pair) for dimers A and B, whereas for dimer C it is $S_3$ (the lower state of the exciton-split pair). Thus, dimers A and B can be classified as H-aggregates, and dimer C is a J-aggregate.

The exciton splittings and spectral shifts obtained from spin-scaled ADC(2) calculations are qualitatively similar to those obtained with original ADC(2), and overall blue-shift is observed when applying spin-scaling (see Figs. S5, S6 and Tables S2, S3).

**Fig. 6:**
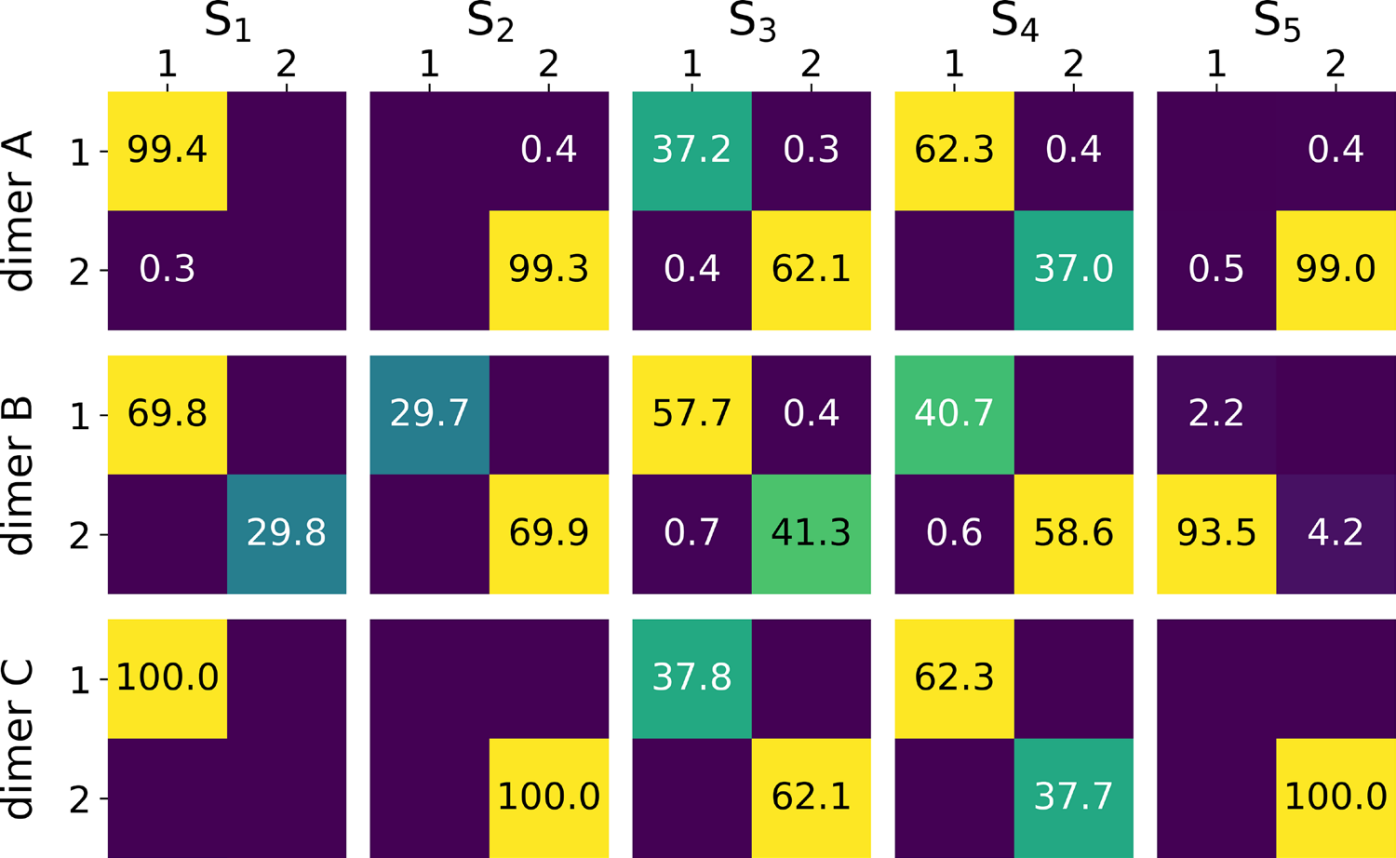
FTDMs for dimers. The FTDM matrices (shown as heat maps) for the lowest five transitions of dimers A (top row), B (middle row), and C (bottom row), computed at the TD-$\omega$B97X-D level. The tick labels “1” and “2” refer to the individual molecules within the respective dimer (*cf.* Fig. 2b). The FTDM matrix elements (in %) are given (if larger than 0.3%).

Further, to characterize the composition of the dimer exciton states, we computed FTDM matrices for the lowest five excited states of the dimers (Fig. 6). The first observation is that $F_{11} \ne F_{22}$ for all considered states, and all dimers. This is in contrast to the simple exciton model, in which each monomer contributes 50% to the exciton state (eigenvector components are $\pm \frac{1}{\sqrt{2}}$)^18^. This asymmetry underlines the importance of molecular orientation for the composition of exciton states. Specifically, the $n\pi ^*$ states $S_1$ and $S_2$ are completely localized on either monomer ($F_{XX} \approx 100\%$) for dimers A and C. For dimer B, these states are partially localized with the $\sim$70%:30% composition. The $\pi \pi ^*$ states $S_3$ and $S_4$ are more delocalized, showing the $\sim$60%:40% ratio of diagonal FTDM elements. Finally, we note that charge transfer contributions (off-diagonal elements $F_{XY},\ X\ne Y$) are small ($<1\%$) for states $S_1$–$S_4$. However, $F_{21}\approx 94\%$ for $S_5$ of dimer B, demonstrating that $S_5$ is a CT state in this case. The corresponding NTOs are shown in Fig. S7.

### Larger aggregates

In this section, we study larger aggregates of methoxy-AAP1, including tetramers ($N=4$, Fig. 2a, c), octamers ($N=8$, Fig. 2d, f), hexadecamers ($N=16$, Fig. 2e, f) and a dotriacontamer ($N=32$, Fig. 2f). We calculated their absorption spectra using TD-$\omega$B97X-D/def2-SV(P). We stress that we use here the “supermolecular” approach, *i.e.*, the whole aggregate is considered as one (large) molecule, and no exciton-type approximations are applied.

**Fig. 7:**
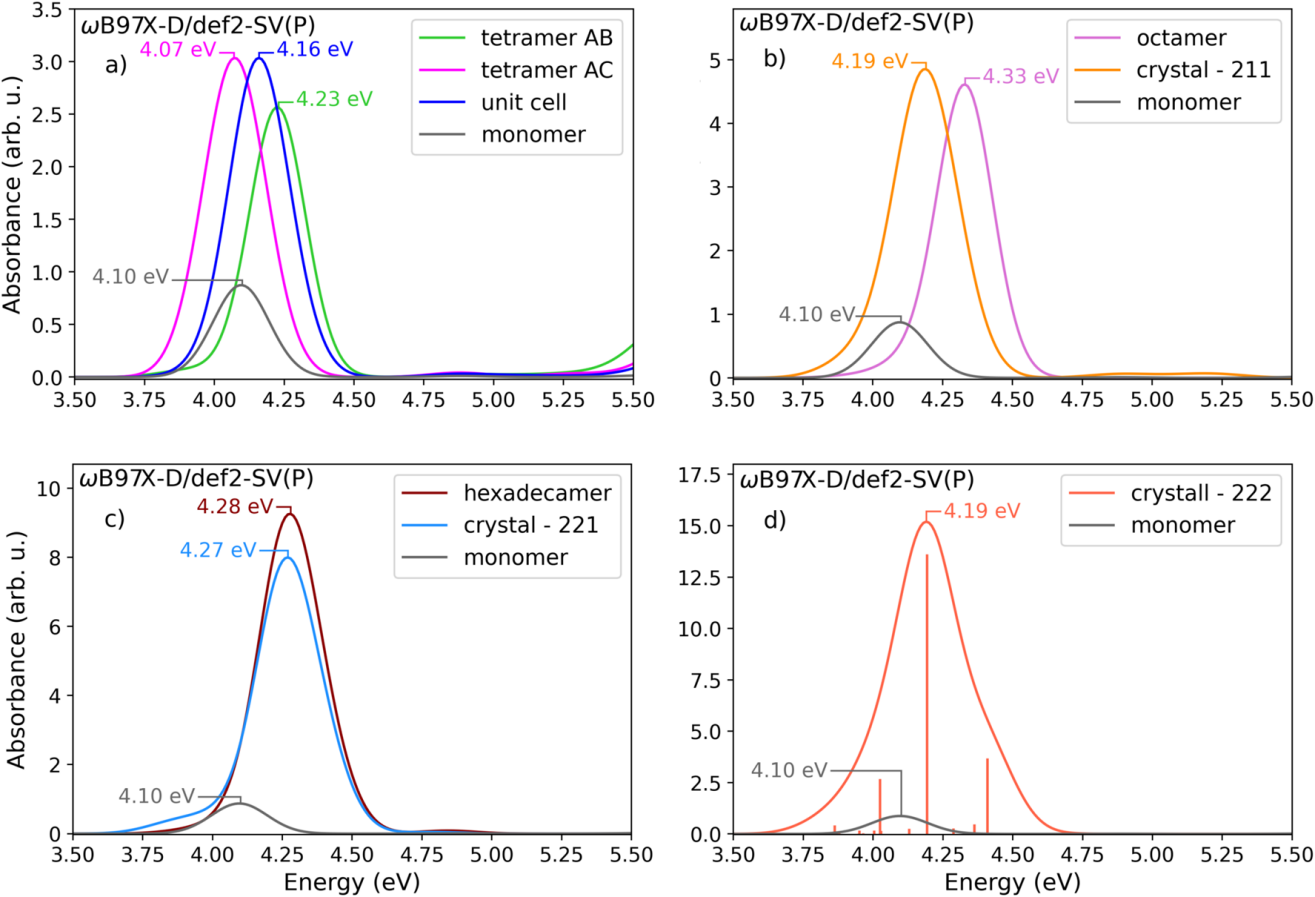
Spectra of larger aggregates. The $\pi \pi ^*$ absorption bands of the larger aggregates of methoxy-AAP1 calculated with TD-$\omega$B97X-D/def2-SV(P). (**a**) Tetramers ($N=4$), (**b**) octamers ($N=8$), (**c**) hexadecamers ($N=16$), (**d**) dotriacontamer ($N=32$). The monomer spectrum is shown in all panels for comparison. The peak positions are provided. In panel (**d**), also the stick spectrum for the dotriacontamer is shown.

The spectra of three considered tetramers (tetramer AB, tetramer AC, and “unit cell”) are shown in Fig. 7a together with the monomer spectrum. The $\pi \pi ^*$ absorption band is blue-shifted for tetramer AB and the unit cell, whereas it is slightly red-shifted for tetramer AC, all in comparison to the monomer absorption band. The spectral shifts calculated from the peak positions of the broadened spectra are +0.13 eV, +0.06 eV and −0.03 eV for tetramer AB, the unit cell, and tetramer AC, respectively.

The brightest states and the exciton splittings as well as the spectral shifts (calculated either as differences of peak positions or using the excitation energies of the brightest states) are collected in Table 3. The $\pi \pi ^*$ exciton splittings of tetramers (*t*) were obtained as $E^{(t)}_{S_8} - E^{(t)}_{S_5}$, because the monomeric $S_1$ ($n\pi ^*$) state splits into four tetrameric states ($S_1$–$S_4$) and the monomeric $S_2$ ($\pi \pi ^*$) state splits into tetrameric $S_5$–$S_8$ states. The exciton splittings of the tetramers vary from 0.18 eV (tetramer AC) to 0.37 eV (tetramer AB). The brightest states are $S_8$ for tetramer AB, and $S_7$ for tetramer AC and the unit cell. The FTDM matrices for the tetramer $n\pi ^*$ states ($S_1$–$S_4$) are shown in Fig. S8, and for the tetramer $\pi \pi ^*$ states ($S_5$–$S_8$) in Fig. S9. The $\pi \pi ^*$ states are more delocalized than the $n\pi ^*$ states. At that, the FTDM diagonal is asymmetric, with a single monomer bearing the highest FTDM element. This is in contrast to the results for model $\pi$-stacked azobenzene tetramers^79^, for which the FTDM diagonal is symmetric. This difference again demonstrates the role of molecular orientation present in realistic geometries in formation of the exciton states. We note that the asymmetry of the FTDM diagonal observed here is preserved when using a DFT-optimized crystal geometry instead of the experimental one (see Fig. S23). We also note that, in general, an exciton localization pattern is determined by the interplay of the exciton coupling and geometrical distortions^80^.

**Table 3:** Spectral characteristics of larger aggregates.

Aggregate	*N*	Brightest state	*E* (eV)	Exciton splitting (eV)	Peak shift (eV)	State shift (eV)
Tetramer AB	4	8	4.23	0.37	0.13	0.13
Tetramer AC	4	7	4.06	0.18	−0.03	−0.03
Unit cell	4	7	4.14	0.30	0.06	0.05
Octamer	8	16	4.33	0.44	0.23	0.23
Crystal-211	8	15	4.17	0.43	0.09	0.07
Hexadecamer	16	30	4.26	0.51	0.18	0.17
Crystal-221	16	31	4.25	0.59	0.17	0.16
Crystal-222	32	61	4.19	0.63	0.09	0.10

The brightest $\pi \pi ^*$ states, corresponding excitation energies (*E*), exciton splittings, and spectral shifts for larger aggregates (composed of *N* molecules), calculated using TD-$\omega$B97X-D/def2-SV(P).

The spectra of the linear octamer and 3D crystal-211 (both systems consist of $N=8$ molecules) are shown in Fig. 7b. Both show a blue shift with respect to the monomer, with the linear octamer demonstrating the largest blue shift (0.23 eV) among the models considered in this work (Table 3). Moreover, for the octamer, the brightest state is $S_{16}$, which is equal to 2*N*. This agrees with its H-aggregate nature. In contrast, for crystal-211, the brightest state is $S_{15}$ ($2N-1$) and the blue shift is much smaller, 0.07 eV (Table 3). The exciton splitting of both systems, calculated as $E^{(o)}_{S_{16}} - E^{(o)}_{S_9}$ (*o* stands for $N=8$), is $\sim$0.4 eV (Table 3). The FTDM matrices for states $S_1$–$S_{16}$ of the linear octamer are shown in Fig. S10. They are mostly diagonal, demonstrating that the CT contributions are small. The brightest state of the octamer, $S_{16}$, is rather delocalized. For crystal-211, the FTDM matrices are presented in Fig. S11. It is seen that each $n\pi ^*$ state is well localized in this case, in contrast to the linear octamer (compare Figs. S10 and S11).

The spectra of systems with $N=16$ (hexadecamer and crystal-221) are shown in Fig. 7c. The peak positions are similar, 4.27 eV for crystal-211 and 4.28 eV for the hexadecamer. Comparing the latter with the octamer, we observe that the peak position is red-shifted by 0.05 eV. The second row of molecules displaced along the C axis creates a J-aggregate on top of the H-aggregation present in the octamer, thus reducing the blue shift. Indeed, the brightest state of the hexadecamer is $S_{30}$ ($2N-2$), not $S_{32}$ (2*N*), see Table 3. For crystal-221, the brightest state is $S_{31}$ ($2N-1$). In this case, again both H- and J-aggregation modes occur; the former in the AB plane and the latter along the C direction. The exciton splittings $E^{(h)}_{S_{32}} - E^{(h)}_{S_{17}}$ (with *h* denoting $N=16$) are $\sim$0.5 eV for the hexadecamer and $\sim$0.6 eV for crystal-221 (Table 3). The FTDM matrices for states $S_1$–$S_{32}$ of the systems with $N=16$ are shown in Figs. S12 and S13. Again, they are diagonal, showing that the excitons consist of local excitations. Moreover, similarly to the case of octamers, we observe more localization for the $n\pi ^*$ states of crystal-221 than for the $n\pi ^*$ states of the hexadecamer.

Finally, we calculated the spectrum of crystal-222 — a dotriacontamer ($N=32$) — the largest cluster model considered in this work, see Fig. 7d. The $\pi \pi ^*$ peak position is 4.19 eV, blue-shifted by 0.09 eV with respect to the monomer. The brightest state is $S_{61}$ ($2N-3$). Again, the interplay of H- and J-aggregation occurs here and results in a moderate spectral shift ($\sim$0.1 eV), as for the crystal-211 model ($N=8$). The exciton splitting $E^{(do)}_{S_{64}} - E^{(do)}_{S_{33}} = 0.63$ eV (*do* means $N=32$). This is the largest splitting among all considered models. We note that for an infinitely large crystal, the exciton bandwidth can be roughly estimated as $4\sum _{i=1}^3 \Delta _i$ with $\Delta _i$ being the nearest-neighbor exciton coupling in direction *i*^17^. Using exciton splittings for dimers A, B, C from Table 1, and calculating the corresponding exciton couplings as half exciton splittings, we obtain $4\times (0.06+0.12+0.035)=0.86$ eV, which is somewhat larger than the TD-DFT exciton splitting for crystal-222 (0.63 eV).

**Fig. 8:**
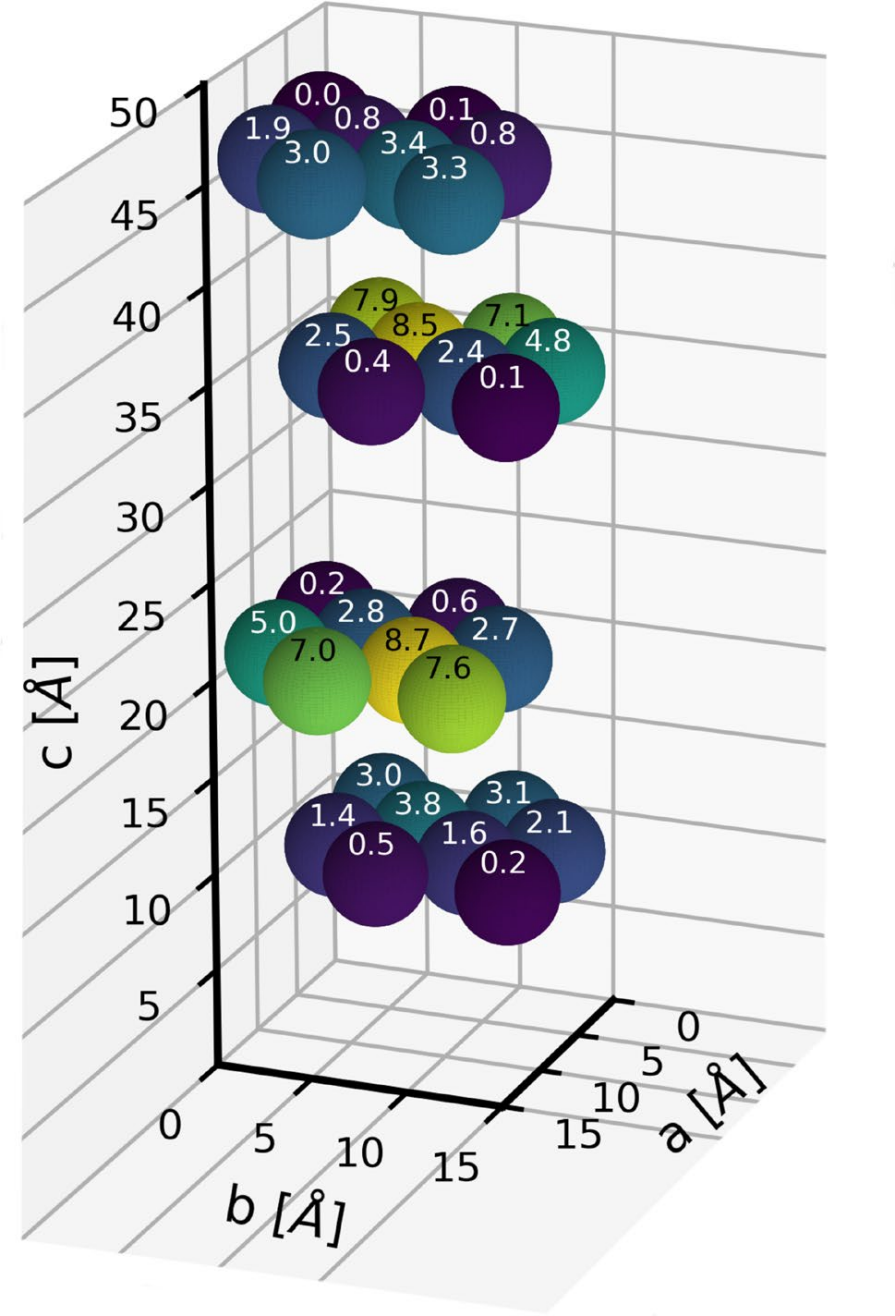
Spatial mapping of FTDM for crystal-222. The diagonal FTDM matrix elements for the brightest state ($S_{61}$) of crystal-222 are shown as spheres positioned at the coordinates of oxygen atoms. The sphere colors correspond to the FTDM values, which are provided in %.

The FTDM matrices for states $S_1$–$S_{64}$ of crystal-222 are shown in Figs. S14 and S15. They are diagonal; the $\pi \pi ^*$ excitons are way more delocalized than the $n\pi ^*$ excitons. The latter are mostly localized on individual monomers. The spatial mapping of the FTDM diagonal for the brightest $\pi \pi ^*$ state ($S_{61}$) is shown in Fig. 8. In this 3D plot, the spheres are placed at the positions of oxygen atoms, and their color and the given numbers visualize the $F_{XX}$ matrix elements, which, in turn, show monomer contributions to the exciton state. As can be seen, the exciton is mostly concentrated in the middle of the aggregate (see yellowish and greenish colors in the second and third rows in Fig. 8). The spatial mapping for the states surrounding the brightest state ($S_{60}$–$S_{64}$) is provided in Fig. S16, demonstrating the exciton structure in real space.

**Fig. 9:**
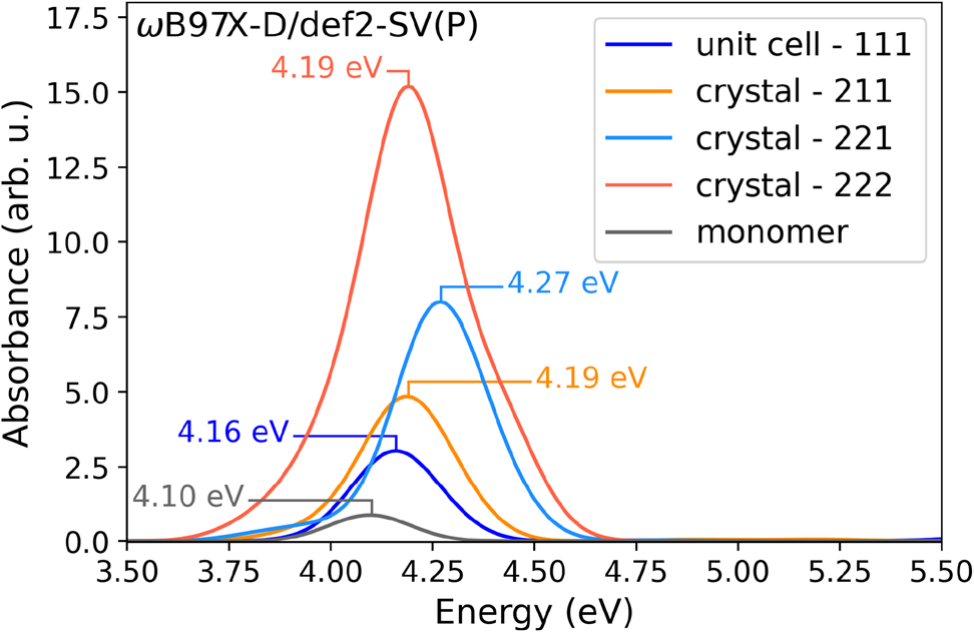
Spectra of crystal models. The $\pi \pi ^*$ absorption bands of different crystal models (including 1, 2, 4, and 8 unit cells) calculated using TD-$\omega$B97X-D/def2-SV(P). The monomer spectum is also shown. The peak positions are provided.

We summarize the absorption spectra of the 3D aggregates in Fig. 9. One can see that the intensity (absorbance) grows with the number of molecules (*N*). Specifically, maximal values (at peak positions) are $\approx$ 0.9, 3.0, 4.9, 8.0, 15.2 for the monomer ($N=1$), unit cell ($N=4$), crystal-211 ($N=8$), crystal-221 ($N=16$), crystal-222 ($N=32$), respectively. The linear fitting of these intensities (without the monomer point) yields a phenomenological law $0.43N+1.28$ (see Fig. S17). As to the peak positions, the blue shift grows up to $N=16$ (reaching 0.17 eV), which is followed by a decrease for $N=32$ (0.09 eV). As already noted above, the spectral shift for crystal-222 ($N=32$) is virtually the same as for crystal-211 ($N=8$).

### Periodic calculations

**Fig. 10:**
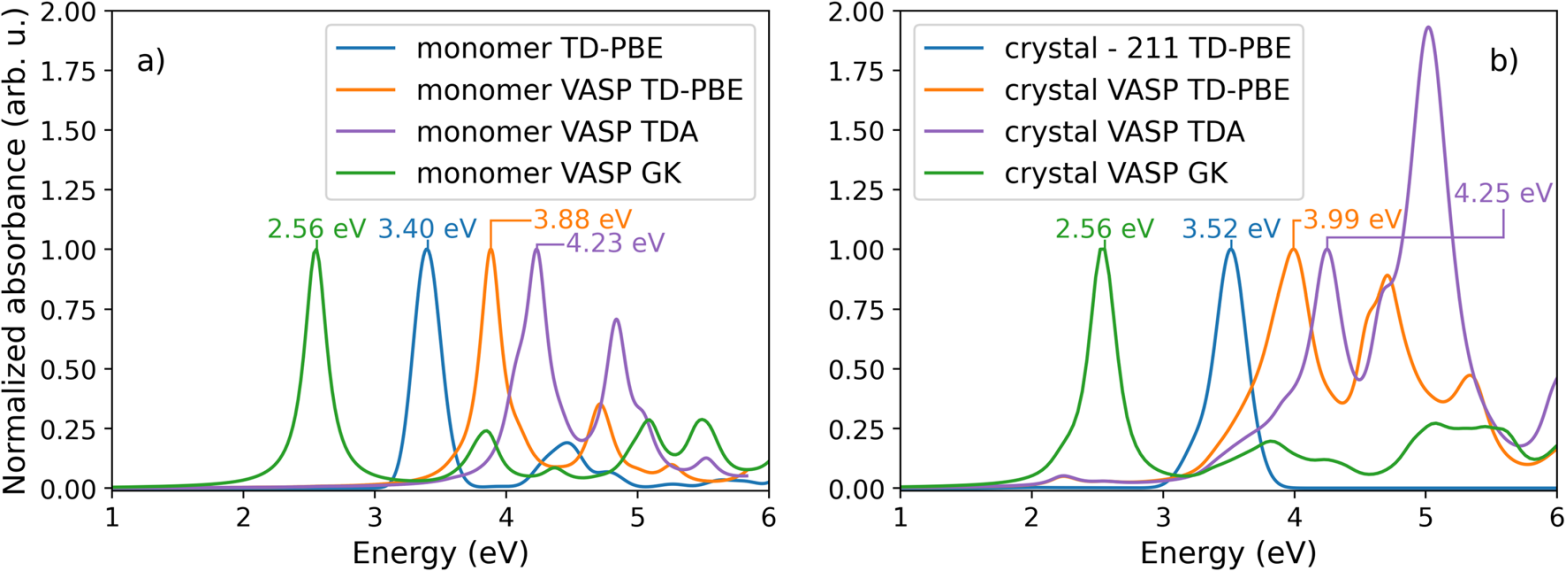
Spectra from periodic calculations. Normalized absorption spectra of (**a**) monomer and (**b**) crystal of methoxy-AAP1 obtained from the periodic VASP calculations (using GK, TDA, and TD approaches and the PBE functional). For comparison, the spectra from “cluster” Gaussian calculations at the TD-PBE/def2-SV(P) level are shown for the monomer in (**a**) and crystal-211 in (**b**). The most intense peak was normalized to 1 in all cases except for VASP TDA in (**b**).

We have also performed periodic DFT calculations with VASP to obtain the absorption spectrum of the crystal (bulk). In these calculations, the PBE functional was used, and three methods were employed to compute optical properties: GK, TD-DFT and TDA (as described in section “Methods”). For comparison, we also performed cluster TD-PBE/def2-SV(P) calculations for the monomer and crystal-211 (see Fig. 2f) using Gaussian. The obtained spectra are shown in Fig. 10.

The crystal-211 model was chosen for cluster calculations (performed with Gaussian) for two reasons: (i) many excited states should be computed to reach the bright $\pi \pi ^*$ transition of the aggregate using PBE (much more states than with $\omega$B97X-D) because of the spurious charge-transfer states observed with semilocal DFT and (ii) at the $\omega$B97X-D level, crystal-211 demonstrated the same spectral shift of 0.09 eV as the largest cluster model (crystal-222). Regarding (i), the brightest state for crystal-211 is $S_{203}$ at the TD-PBE/def2-SV(P) level. At the TD-PBE/def2-SV(P) level, the monomer-to-aggregate shift is 0.12 eV (see Table 4). We note that the PBE spectra are expectedly red-shifted in comparison to the TD-$\omega$B97X-D spectra in Fig. 7b. Specifically, the TD-PBE peak positions are 3.40 eV for the monomer and 3.52 eV for crystal-211 (Fig. 10 and Table 4). At the TDA-PBE level, the spectra are blue-shifted and the monomer-to-aggregate shift is 0.07 eV (for crystal-211, calculated with Gaussian; see Table 4).

**Table 4:** Peak positions and shifts obtained with PBE.

Method	Monomer peak (eV)	Crystal peak (eV)	Peak shift (eV)
Gaussian TD-PBE	3.40	3.52	0.12
Gaussian TDA-PBE	3.65	3.72	0.07
VASP TD-PBE	3.88	3.99	0.11
VASP TDA-PBE	4.23	4.25	0.02
VASP GK-PBE	2.56	2.56	0.00

The $\pi \pi ^*$ peak maxima of the methoxy-AAP1 monomer and crystal structure, and the peak shift (crystal minus monomer). Periodic methods are compared with the “cluster” TD(A)-PBE approach.

Turning to the spectra obtained from the periodic DFT calculations (performed with VASP), we see that the GK method predicts even lower energy for the absorption peak, 2.56 eV (Fig. 10), for both the monomer and the crystal, thus yielding a zero monomer-to-crystal shift (Table 4). The periodic TDA method, in contrast, shows blue-shifted bands with peak positions of 4.23 eV for the monomer and 4.25 eV for the crystal. The corresponding peak shift is 0.02 eV, which is again close to zero. [We note, however, that this shift depends on the number of included occupied and unoccupied bands (see Table S4).] Finally, the full periodic TD-DFT predicts 3.88 eV for the monomer and 3.99 eV for the crystal. The monomer-to-crystal shift is 0.11 eV in this case, which is close to the result obtained with the cluster calculation (0.12 eV). We note, however, that the absolute peak positions are rather different for the cluster and periodic TD-DFT calculations. For example, for the monomer, the cluster approach predicts 3.40 eV, whereas the periodic approach yields 3.88 eV. The blue-shifted monomer absorption observed in the periodic calculations may be explained by a small number of occupied and virtual bands used for the monomer (to be consistent with the calculations on the crystal). If more bands are included, the peak is red-shifted to around 3.35 eV (see Fig. S19). Periodic calculations using TD-B3LYP show a qualitatively similar trend for the monomer peak, as well as an expected blue-shift of the peak positions compared to TD-PBE (Figs. S20, S21). However, the monomer-to-crystal peak shift (0.09 eV; see Table S4) is very similar to the TD-PBE result. The number of included bands also affects the difference in peak shifts obtained with TDA-PBE compared to TD-PBE (see Table S4). Because the peak shift of the periodic TD-PBE calculation is in good agreement with the cluster TD-PBE calculation and the periodic TD-B3LYP calculation, we consider the TD-DFT results more robust. For completeness, the crystal band structure is provided in Fig. S25 and partial charge densities showing localization of the bands in Fig. S26. The latter demonstrate that valence bands 157–160 are of the $\pi$ character, valence bands 161–164 are of the *n* character, and conduction bands 165–168 show the typical $\pi^*$-type distribution.

While there is no direct experimental reference for the peak shift, the optical spectrum was reported for a crystalline film of methoxy-AAP1 in Ref.^41^. The $\pi \pi ^*$ peak position for the film was found to be 340 nm, whereas for the monomer (in aqueous solution) it is 347 nm^41^. This results in a monomer-to-film shift of $\sim$0.07 eV, which is close to our theoretical estimates of $\sim$0.1 eV.

## Conclusions

In summary, we studied absorption spectra and exciton states of arylazopyrazole aggregates (made of methoxy-AAP1) using first-principles quantum chemical calculations. The aggregate structures were extracted from the experimenally determined crystal structure^41^. We used primarily long-range corrected TD-DFT (with the $\omega$B97X-D functional) to calculate the excited states of the aggregates of varying size, ranging from $N=2$ (dimers) to $N=32$ (dotriacontamer) molecules. For the dimers, also ADC(2) and its spin-scaled variants were tested. Moreover, periodic DFT and TD-DFT calculations (with the PBE and B3LYP functionals) were performed for the crystal structure of methoxy-AAP1.

The fraction of transition density matrix (FTDM) analysis was used to characterize composition of the exciton states (contributions of local (LE) and charge transfer (CT) excitations). We found that the lowest excited states of the studied aggregates, arising from the $S_1$ ($n\pi ^*$) and $S_2$ ($\pi \pi ^*$) monomer states, are predominantly made of LE (thus being Frenkel exciton states), whereas CT contributions are small. The FTDM analysis demonstrated that the $n\pi ^*$ excitons are more localized than the $\pi \pi ^*$ excitons. Considering all aggregates, the largest predicted $\pi \pi ^*$ exciton splitting is 0.63 eV, observed for the crystal-222 model ($N=32$) — the largest considered cluster model in this work. Among dimers, the largest splitting is 0.24 eV, found for dimer B (which is the H-type aggregate). Both blue and red monomer-to-aggregate spectral shifts were observed depending on the mode of aggregation. The largest blue shift, 0.23 eV, was found for the linear octamer model (H-aggregate), whereas the largest red shift, which is only −0.03 eV, was observed for tetramer AC (predominantly J-aggregate). From the periodic TD-DFT calculations, we estimated the monomer-to-crystal shift to be $\sim$0.1 eV, which is close to the experimental monomer-to-film shift ($\sim$0.07 eV)^41^. Taken together, our findings provide new insights into the electronic spectroscopy of the arylazopyrazole aggregates and crystals.

Finally, we note that conformational disorder and excited-state dynamics are expected to affect exciton (de)localization^79,81–84^. Studying these aspects for the arylazopyrazole aggregates is a worthwhile direction for future research. In particular, direct simulations of aggregate photodynamics would allow one to gain insight into photochemical and photophysical processes occurring in these systems.

## Supplementary Information

Supplementary Information.

## Data Availability

The data that support the findings of this study are available in the Zenodo repository, DOI: https://doi.org/10.5281/zenodo.20027417.
