# Supplementary material for: Molecular excitons in arylazopyrazole aggregates: a quantum chemical study
Source: Sci Rep. 2026 Jun 10;16:18029. doi: 10.1038/s41598-026-53659-1 (PMC13254293; doi:10.1038/s41598-026-53659-1)
Supplement: Supplementary file 1 — Supplementary Information. [file 41598_2026_53659_MOESM1_ESM.pdf]

Supporting Information for "Molecular Excitons in Arylazopyrazole Aggregates: A Quantum Chemical Study"

Anna Zehle, Christopher Penschke, and Evgenii Titov*

University of Potsdam, Institute of Chemistry, Theoretical Chemistry, Karl-Liebknecht-Straße 24-25, 14476 Potsdam, Germany

E-mail: titov@uni-potsdam.de

Contents

S1 Monomers **S2**

S2 Dimers **S4**

S2.1 Transition dipole moment S4

S2.2 ADC(2) calculations S5

S2.3 Natural Transition Orbitals (NTOs) S7

S3 Larger Aggregates **S8**

S3.1 Tetramers S8

S3.2 Octamers S9

S3.3 Hexadecamers S11

S3.4 Dotriacontamer S13

S4 Periodic calculations **S16**

S4.1 Spectra obtained using different numbers of bands S16

S4.2 B3LYP calculations S17

S4.3 Comparison of peak positions S18

S5 Further results **S19**

S1 Monomers

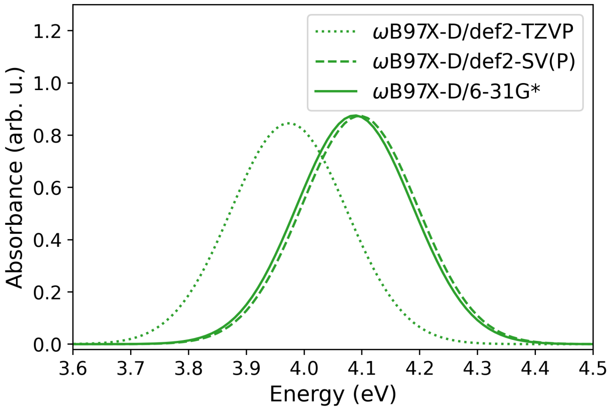

Figure S1: The TD-ωB97X-D spectra of the methoxy-AAP1 monomer calculated at the experimental geometry using different basis sets.

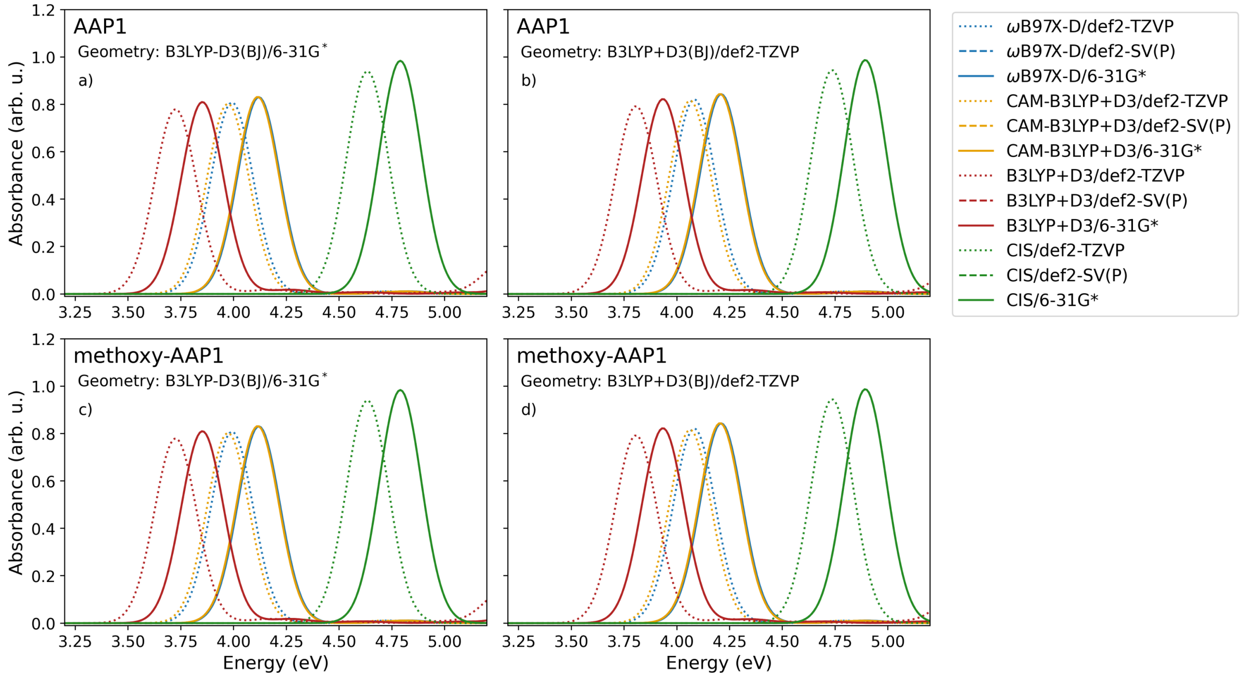

Figure S2: The absorption spectra of AAP1 (a,b) and methoxy-AAP1 (c,d), calculated with various methods at the B3LYP+D3(BJ)/6-31G* geometry (a,c) and at the B3LYP+D3(BJ)/def2-TZVP geometry (b,d).

Table S1: Peak positions for spectra shown in Fig. S2 for both AAP1 and m ethoxy-AAP1, at the geometries optimized using B3LYP-D3(BJ) with either the 6-31G* basis set (denoted as "$631G$") or the def2-TZVP basis set (denoted as "$def2$").

	AAP1		methoxy-AAP1	
Method	E_{631G} (eV)	E_{def2} (eV)	E_{631G} (eV)	E_{def2} (eV)
TD-ωB97X-D/def2-TZVP	3.99	4.08	3.84	3.93
TD-ωB97X-D/def2-SV(P)	4.12	4.21	3.96	4.05
TD-ωB97X-D/6-31G*	4.12	4.21	3.96	4.05
TD-CAM-B3LYP/def2-TZVP	3.97	4.06	3.82	3.90
TD-CAM-B3LYP/def2-SV(P)	4.11	4.21	3.95	4.04
TD-CAM-B3LYP/6-31G*	4.11	4.21	3.95	4.04
TD-B3LYP/def2-TZVP	3.73	3.81	3.54	3.62
TD-B3LYP/def2-SV(P)	3.85	3.93	3.66	3.73
TD-B3LYP/6-31G*	3.85	3.93	3.66	3.73
CIS/def2-TZVP	4.63	7.48	4.54	4.65
CIS/def2-SV(P)	4.79	4.89	4.69	4.80
CIS/6-31G*	4.79	4.89	4.69	4.80

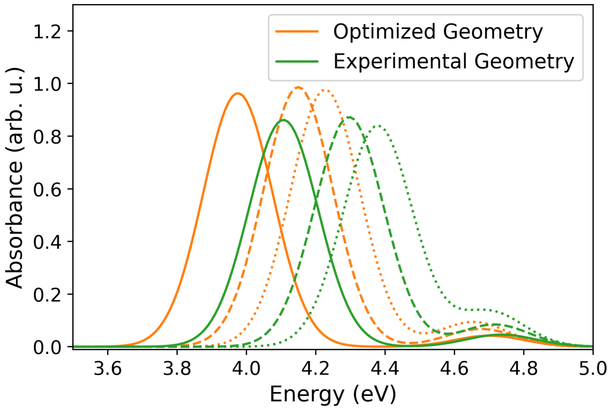

Figure S3: The ADC(2), SCS-ADC(2), and SOS-ADC(2) spectra of the methoxy-AAP1 monomer. The orange curves are based on the geometry optimized at the B3LYP+D3(BJ)/6-31G* level, whereas the green curves correspond to the experimentally determined molecular structure. Dashed lines indicate results obtained with SCS-ADC(2)/cc-pVDZ, dotted lines with SOS-ADC(2)/cc-pVDZ spectra, and solid lines with ADC(2)/cc-pVDZ.

S2 Dimers

S2.1 Transition dipole moment

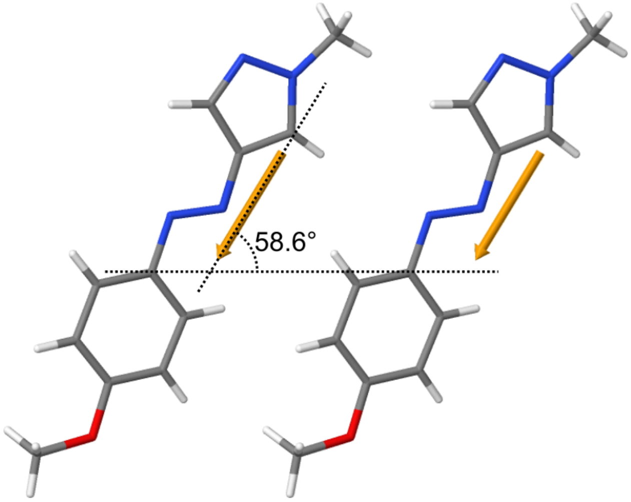

Figure S4: Orientation of monomer transition dipole moments (yellow arrows) in dimer A. The angle formed by the transition dipole moment and intermolecular axis is shown.

S2.2 ADC(2) calculations

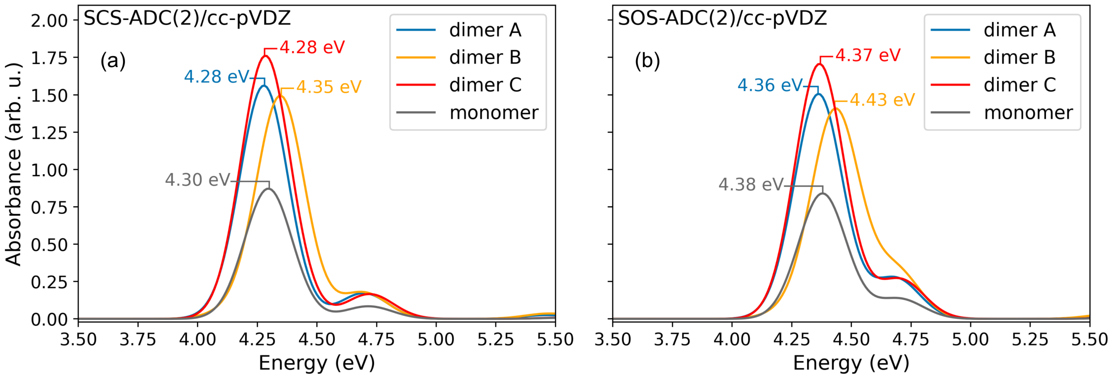

Figure S5: The SCS-ADC(2)/cc-pVDZ (a) and SOS-ADC(2)/cc-pVDZ (b) spectra of dimers A, B, C and the monomer.

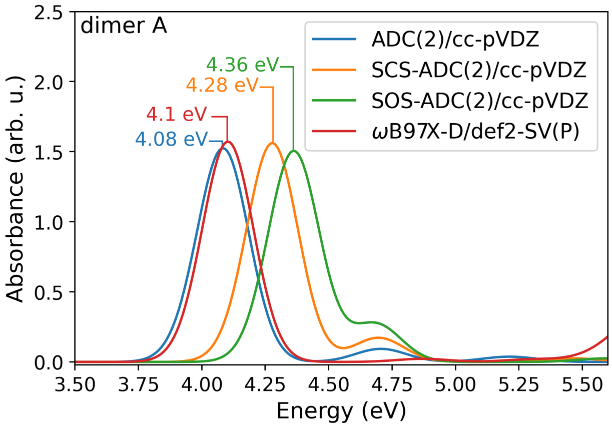

Figure S6: The spectra of dimer A calculated with TD-ωB97X-D and different ADC(2) methods.

Table S2: Exciton splittings and monomer-to-dimer spectral shifts for methoxy-AAP1 dimers A, B, and C calculated with SCS-ADC(2)/cc-pVDZ and SOS-ADC(2)/cc-pVDZ. The "peak shifts" were determined from the maxima of the spectra, whereas the "state shifts" were obtained using excitation energies of the brightest states.

	SCS-ADC(2)/cc-pVDZ			SOS-ADC(2)/cc-pVDZ		
dimer	exciton splitting (eV)	peak shift (eV)	state shift (eV)	exciton splitting (eV)	peak shift (eV)	state shift (eV)
A	0.10	−0.02	−0.02	0.10	−0.02	−0.01
B	0.20	0.05	0.05	0.19	0.05	0.05
C	0.06	−0.02	−0.03	0.06	−0.01	−0.03

Table S3: Excitation energies (E) and oscillator strengths (f) for the lowest five excited states of the methoxy-AAP1 monomer and its dimers, calculated using SCS-ADC(2)/cc-pVDZ and SOS-ADC(2)/cc-pVDZ. The brightest transitions are highlighted in bold.

	monomer		dimer A		dimer B		dimer C	
state	E (eV)	f	E (eV)	f	E (eV)	f	E (eV)	f
	SCS-ADC(2)/cc-pVDZ							
S_1	3.40	0.00	3.38	0.00	3.38	0.00	3.39	0.00
S_2	**4.30**	**0.87**	3.41	0.00	3.38	0.00	3.40	0.00
S_3	4.72	0.08	4.18	0.06	4.15	0.05	**4.27**	**1.38**
S_4	5.51	0.01	**4.28**	**1.53**	**4.35**	**1.49**	4.33	0.44
S_5	5.61	0.00	4.69	0.03	4.67	0.02	4.72	0.08
	SOS-ADC(2)/cc-pVDZ							
S_1	3.47	0.00	3.46	0.00	3.45	0.00	3.46	0.00
S_2	**4.38**	**0.84**	3.48	0.00	3.46	0.00	3.47	0.00
S_3	4.70	0.13	4.26	0.05	4.24	0.04	**4.35**	**1.35**
S_4	5.62	0.01	**4.36**	**1.47**	**4.43**	**1.39**	4.41	0.41
S_5	5.70	0.01	4.68	0.02	4.66	0.02	4.70	0.13

S2.3 Natural Transition Orbitals (NTOs)

$S_0 \rightarrow S_1$	$S_0 \rightarrow S_2$	$S_0 \rightarrow S_3$	$S_0 \rightarrow S_4$	$S_0 \rightarrow S_5$
hole particle	hole particle	hole particle	hole particle	hole particle
Dimer A				
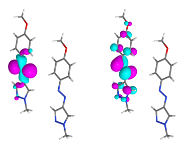	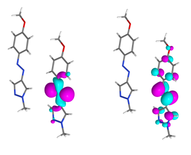	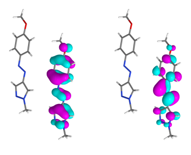	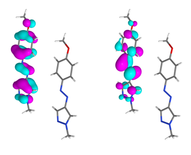	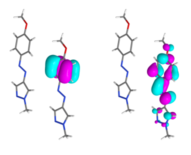
100.5 %	100.5 %	62.0 %	61.8 %	58.8 %
		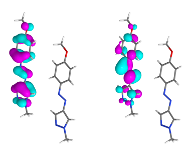	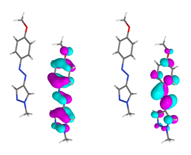	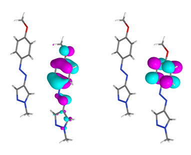
		36.5 %	36.5 %	41.1 %
Dimer B				
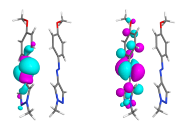	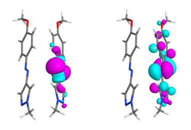	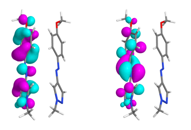	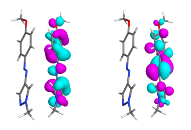	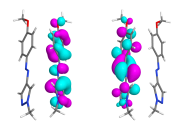
70.5 %	70.5 %	58.0 %	57.8 %	97.3 %
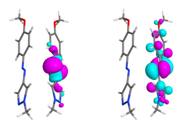	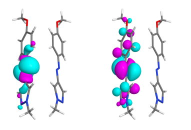	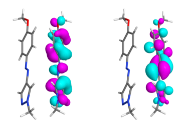	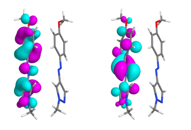	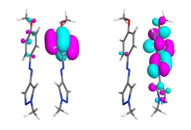
30.0 %	30.0 %	40.6 %	40.5 %	1.3 %
Dimer C				
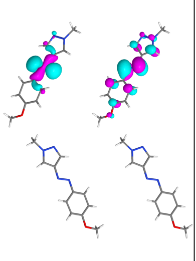	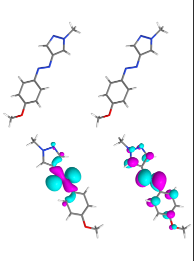	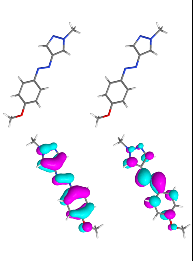	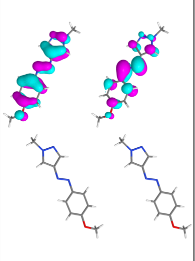	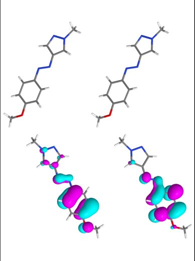
100.5 %	100.5 %	61.3 %	61.2 %	52.7 %
		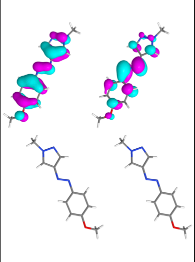	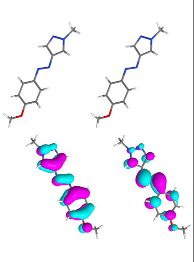	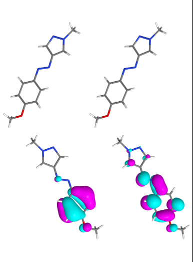
		37.2 %	37.2 %	47.2 %

Figure S7: NTOs for various methoxy-AAP1 dimers, for transitions from $S_0 \rightarrow S_1$ to $S_0 \rightarrow S_5$. The contributions of each NTO pair are provided.

S3 Larger Aggregates

S3.1 Tetramers

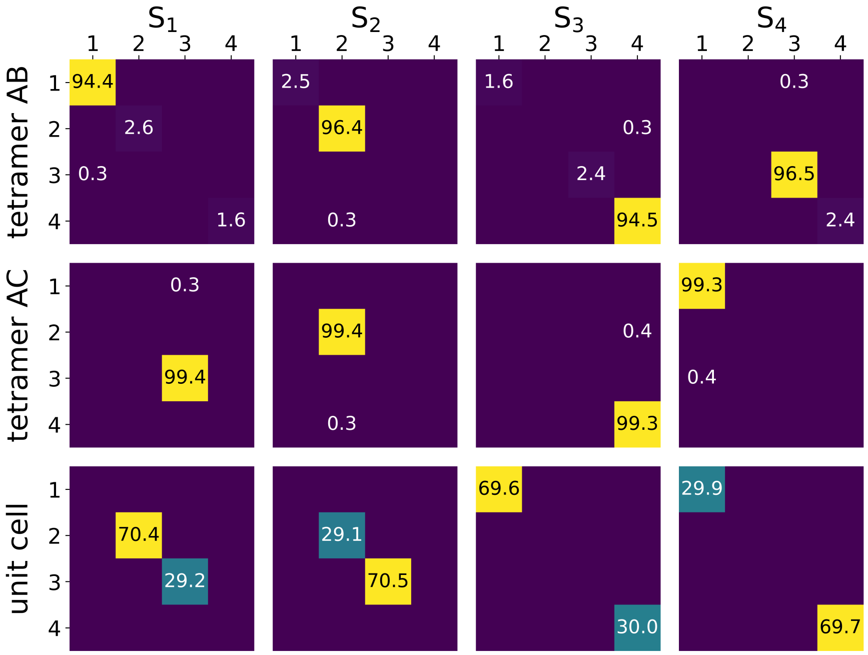

Figure S8: FTDM matrices for the tetramers ($N = 4$), for states S_1–S_4 ($n\pi^*$ states).

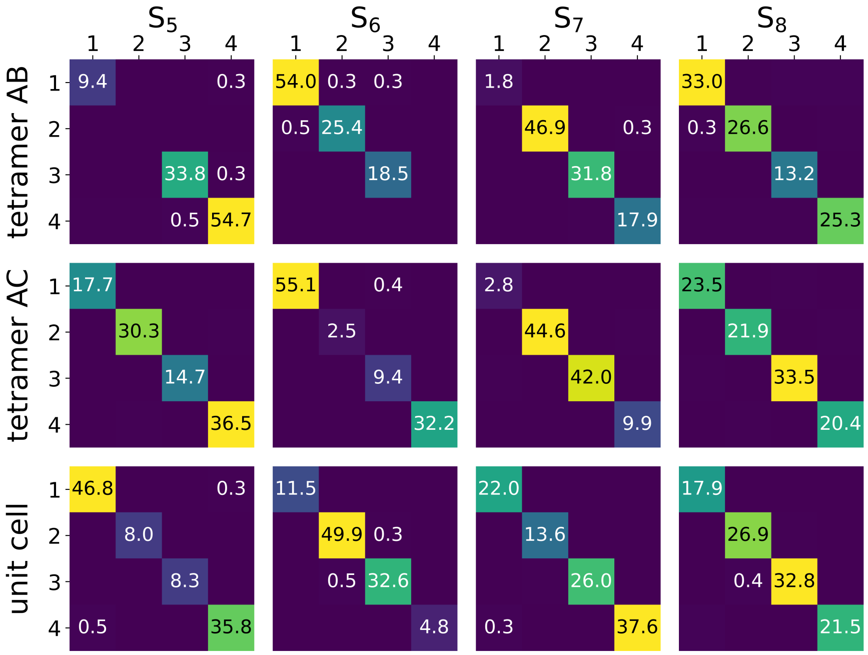

Figure S9: FTDM matrices for the tetramers ($N = 4$), for states S_5–S_8 ($\pi\pi^*$ states).

S3.2 Octamers

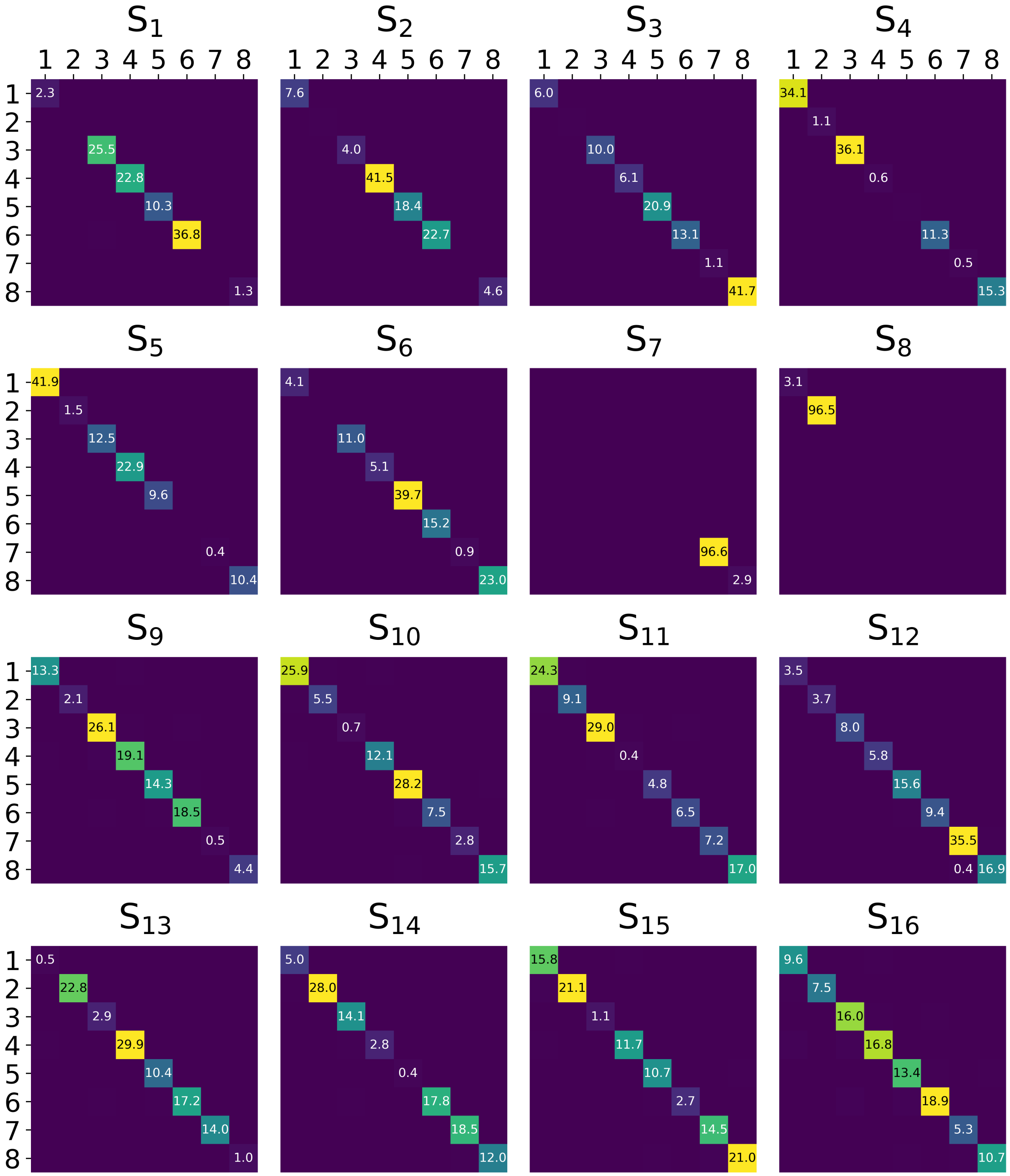

Figure S10: FTDM matrices for the octamer ($N = 8$), for states S_1–S_{16}.

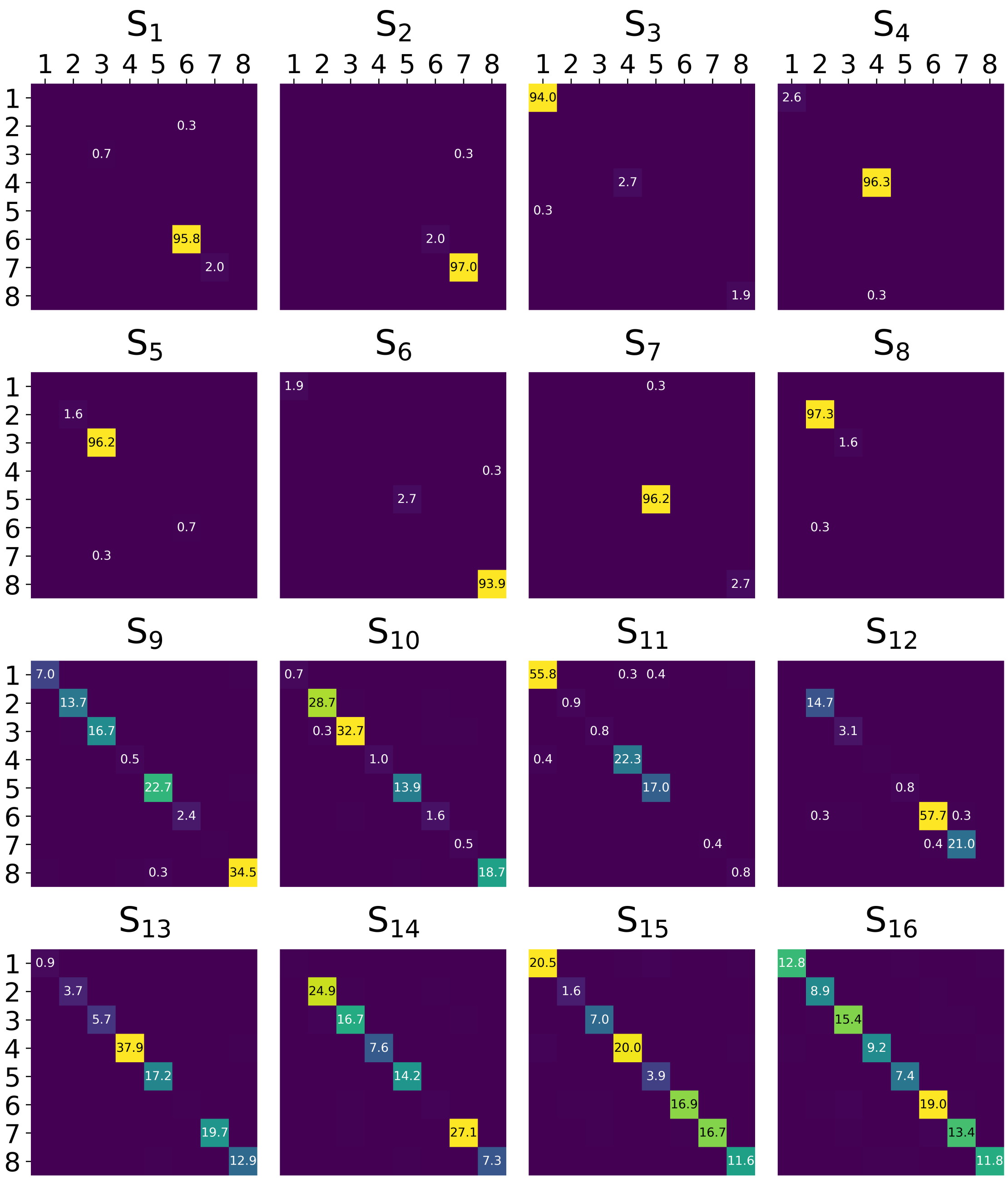

Figure S11: FTDM matrices for crystal-211 ($N = 8$), for states S_1–S_{16}.

S3.3 Hexadecamers

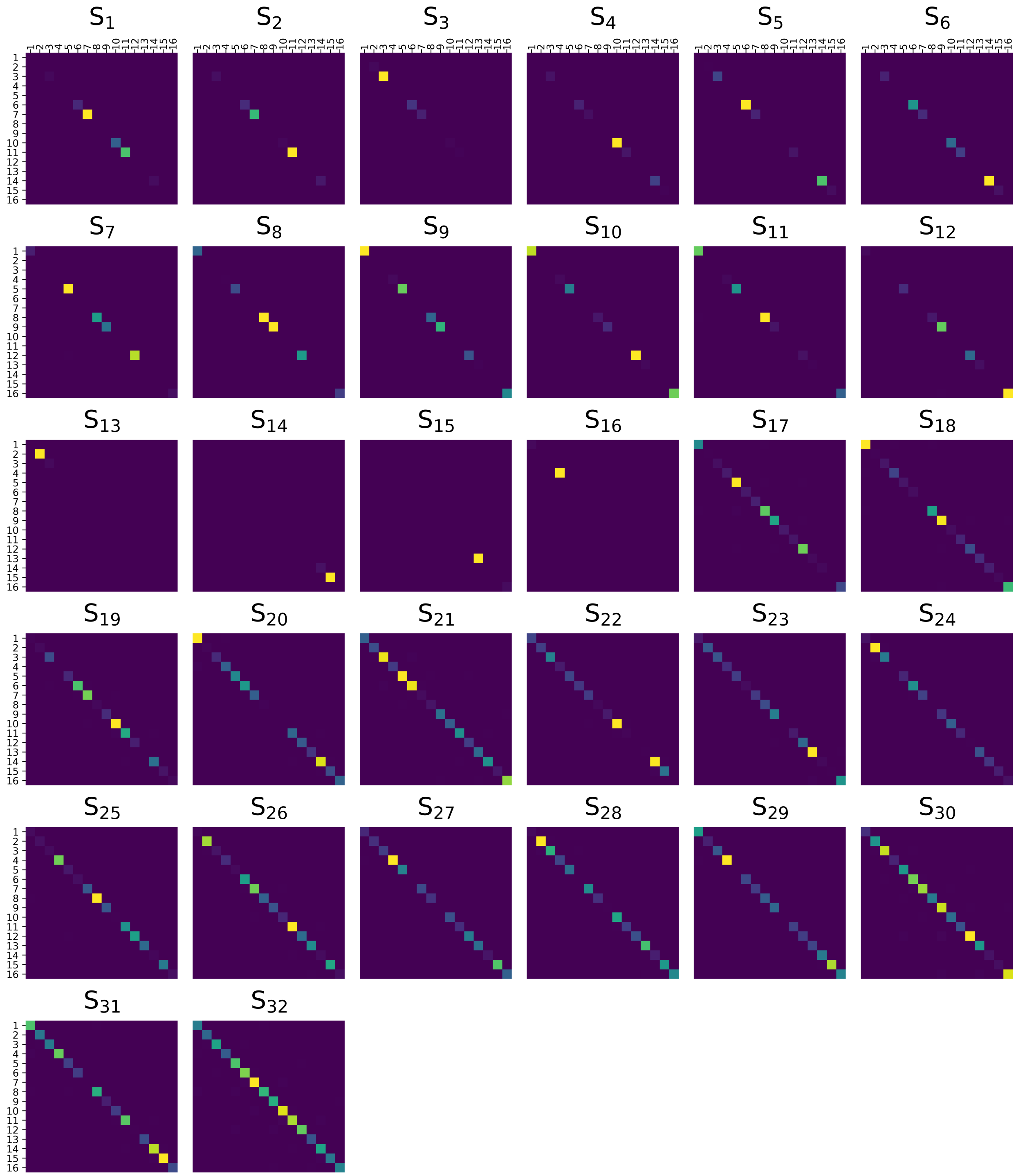

Figure S12: FTDM matrices for the hexadecamer ($N = 16$), for states S_1–S_{32}.

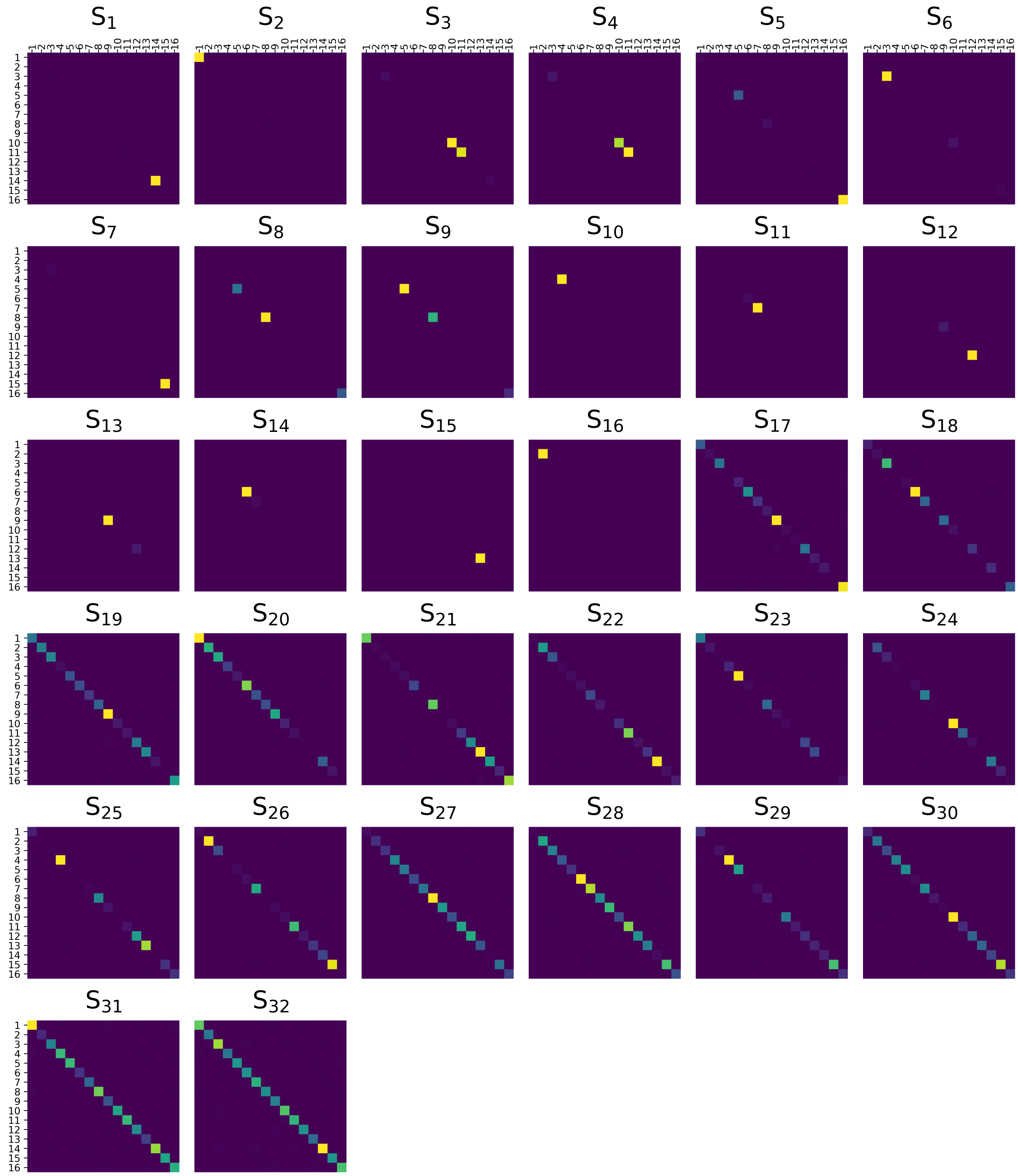

Figure S13: FTDM matrices for crystal-221 ($N = 16$), for states S_1–S_{32}.

S3.4 Dotriacontamer

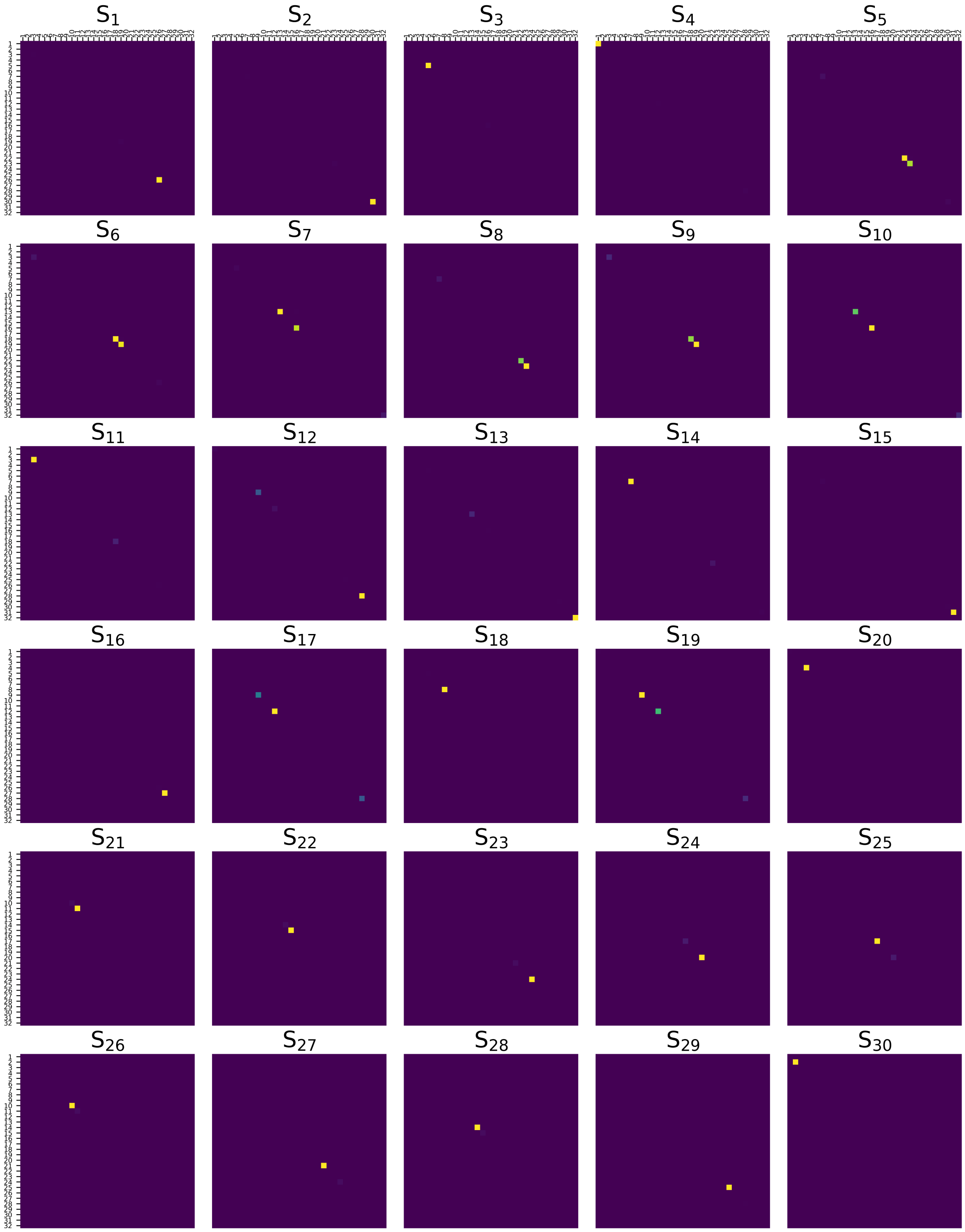

Figure S14: FTDM matrices for the dotriacontamer ($N = 32$), for states S_1–S_{30}.

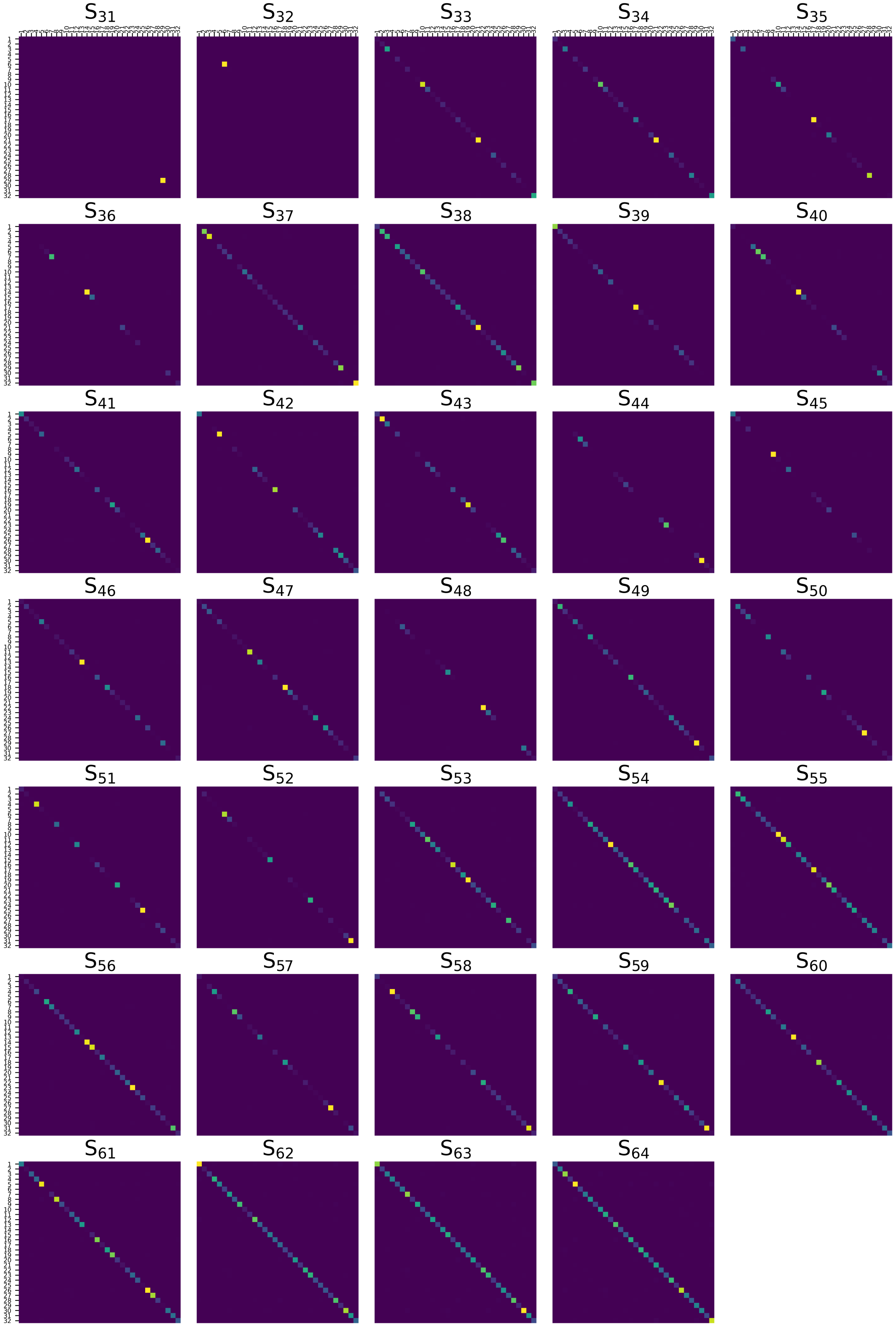

Figure S15: FTDM matrices for the dotriacontamer ($N = 32$), for states S_{31}–S_{64}.

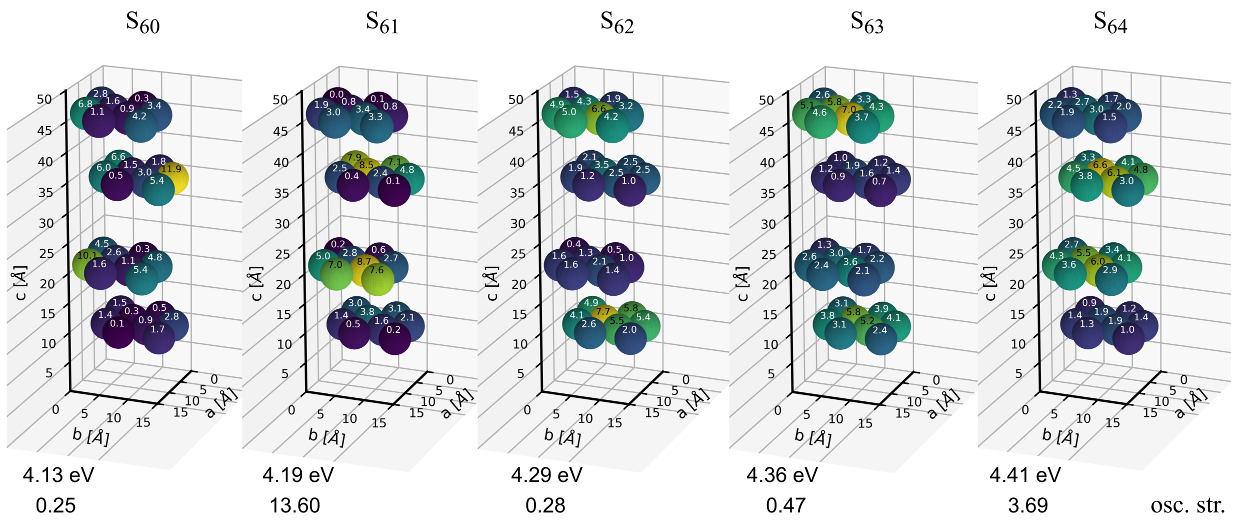

Figure S16: Spatial mapping of FTDM for crystal-222, for states S_{60}–S_{64}. The diagonal FTDM matrix elements are shown as spheres positioned at the coordinates of oxygen atoms. The sphere colors correspond to the FTDM values, which are provided in %.

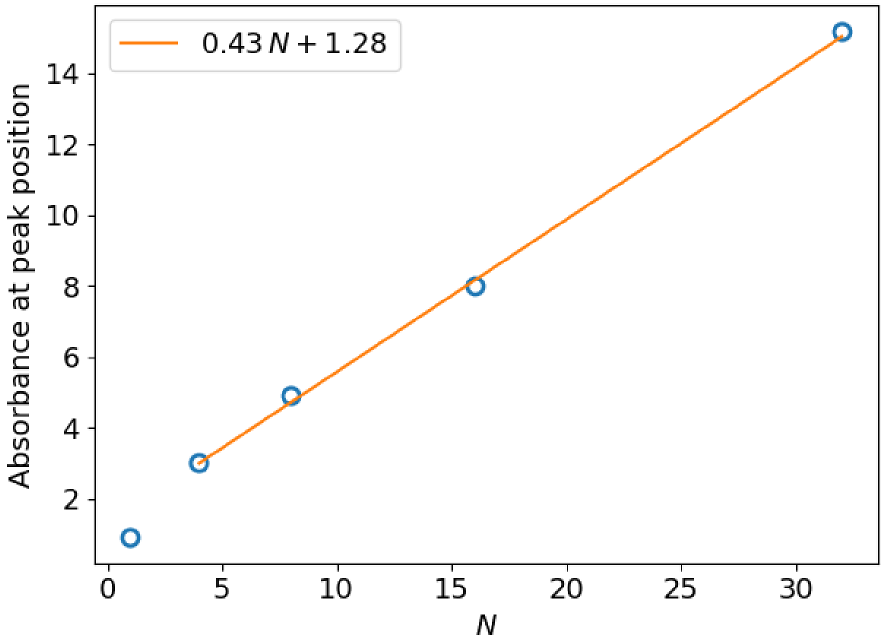

Figure S17: Absorbance at peak position as a function of the number of molecules N, and the linear fit between $N = 4$ and $N = 32$. The corresponding spectra are provided in Fig. 9 of the main text.

S4 Periodic calculations

S4.1 Spectra obtained using different numbers of bands

(a) monomer (b) crystal

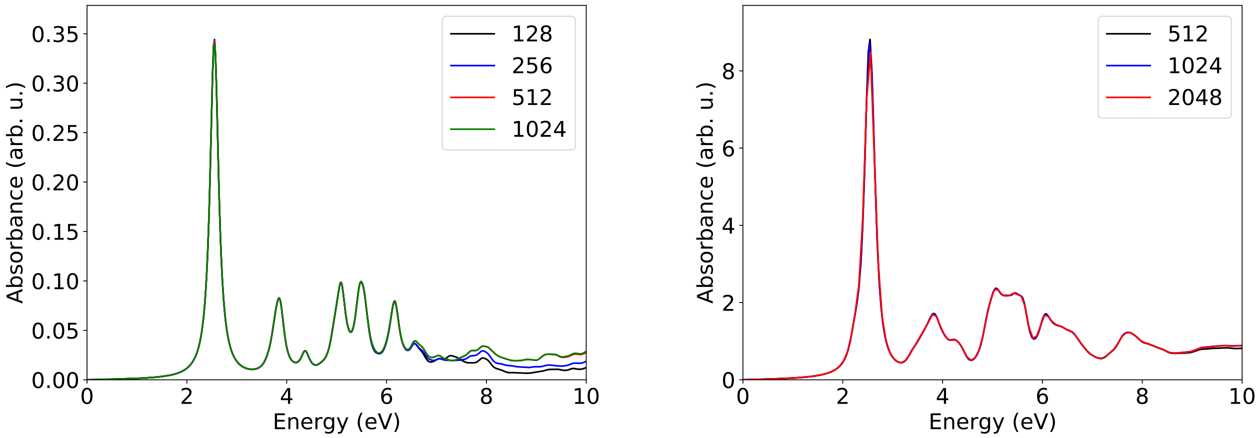

Figure S18: Spectra of methoxy-AAP1 from periodic PBE calculations for (a) the monomer and (b) the crystal using the Green–Kubo (GK) formula. The legend indicates the total number of bands included in the calculation.

(a) TDA (b) TD-DFT

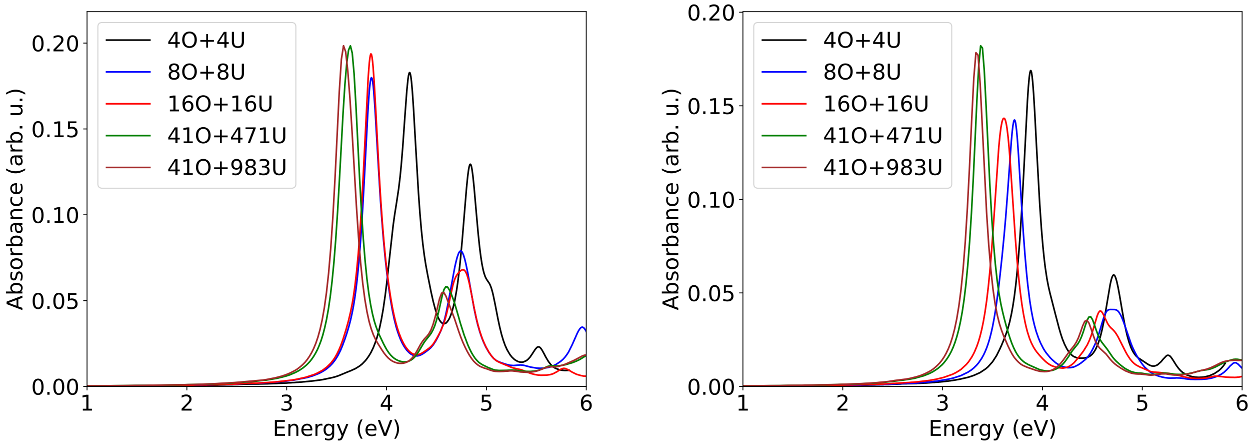

Figure S19: Spectra of methoxy-AAP1 from periodic PBE calculations for the monomer using (a) TDA and (b) TD-DFT. The notation "nO+mU" indicates the number of included occupied ("O") and unoccupied ("U") bands.

S4.2 B3LYP calculations

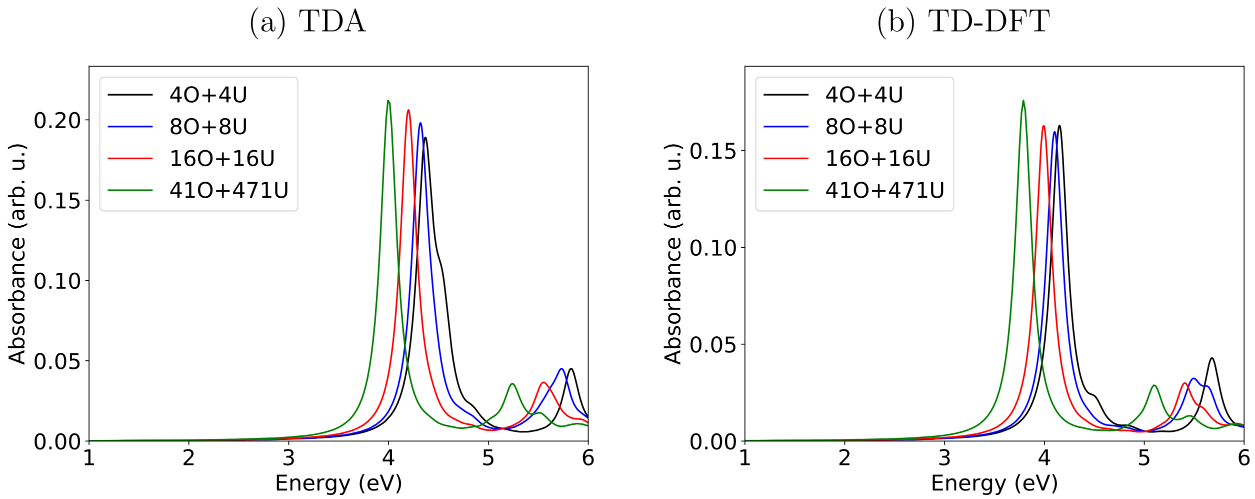

Figure S20: Spectra of methoxy-AAP1 from periodic B3LYP calculations for the monomer using (a) TDA and (b) TD-DFT. The notation "nO+mU" indicates the number of included occupied ("O") and unoccupied ("U") bands.

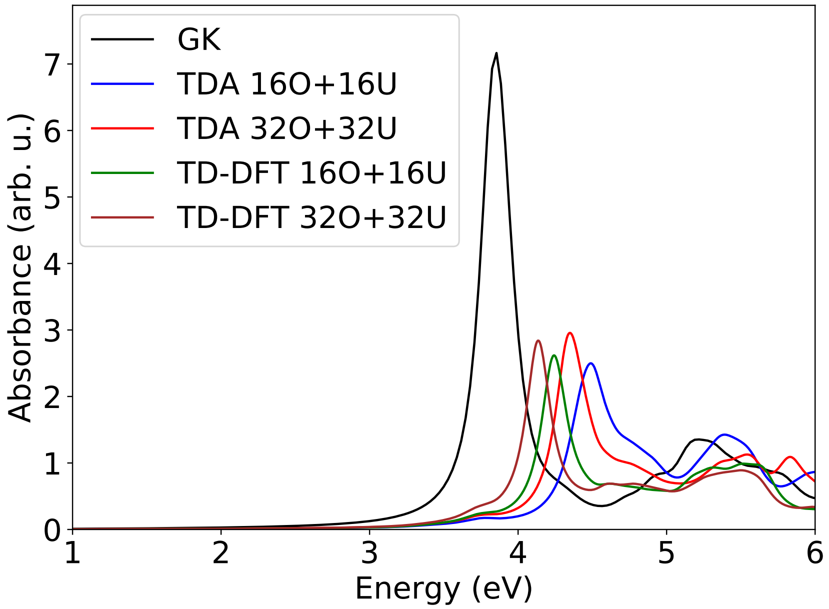

Figure S21: Spectra of methoxy-AAP1 from periodic B3LYP calculations for the crystal. The notation "nO+mU" indicates the number of included occupied ("O") and unoccupied ("U") bands.

S4.3 Comparison of peak positions

The positions of the $\pi\pi^*$ peak in the spectra obtained from periodic calculations are compared in Tab. S4. B3LYP values are systematically blue-shifted compared to PBE for a given method (TDA/TD-DFT/GK), but the qualitative trends are similar: (i) peaks are shifted to higher energies in the sequence GK<TD-DFT<TDA; (ii) peaks are shifted to lower energies upon inclusion of more bands in the TDA and TD-DFT calculations; and (iii) the monomer-to-crystal shift decreases with increasing number of bands in the TDA and TD-DFT calculations. Thus, while the peak positions differ between B3LYP and PBE, the monomer-to-crystal shift is very similar, at least for TD-DFT.

Table S4: Peak positions and shifts obtained with PBE and B3LYP. The $\pi\pi^*$ peak maxima of the methoxy-AAP1 monomer and crystal structure, and the peak shift (crystal minus monomer). The notation "nO+mU" indicates the number of included occupied ("O") and unoccupied ("U") bands per monomer, which means that the calculations for the crystal include four times as many bands. Note that both, PBE and B3LYP results, were obtained using a 4×3×1 k-point grid for the crystal (in contrast to the PBE calculations discussed in the main text and in sec. S4.1, which used a 8×7×2 k-point grid).

method	monomer peak (eV)	crystal peak (eV)	peak shift (eV)
TD-PBE 4O+4U	3.88	3.99	0.11
TD-PBE 8O+8U	3.72	3.74	0.02
TD-PBE 16O+16U	3.61	3.55	−0.06
TDA-PBE 4O+4U	4.23	4.25	0.02
TDA-PBE 8O+8U	3.85	4.08	0.23
TDA-PBE 16O+16U	3.84	3.83	−0.01
GK-PBE	2.56	2.54	−0.01
TD-B3LYP 4O+4U	4.15	4.24	0.09
TD-B3LYP 8O+8U	4.10	4.13	0.03
TDA-B3LYP 4O+4U	4.37	4.49	0.12
TDA-B3LYP 8O+8U	4.32	4.35	0.03
GK-B3LYP	3.90	3.85	−0.04

S5 Further results

Table S5: Excitation energy differences, "TD-ωB97X-D/def2-SV(P) minus ADC(2)/cc-pVDZ", in eV, for the lowest five excited states of the methoxy-AAP1 monomer and its dimers. The brightest transitions are highlighted in bold.

	monomer	dimer A	dimer B	dimer C
S_1	−0.33	−0.31	−0.33	−0.33
S_2	**−0.01**	−0.32	−0.32	−0.33
S_3	0.15	0.01	−0.01	**−0.02**
S_4	0.20	**0.01**	**0.04**	0.00
S_5	0.17	0.12	0.03	0.15

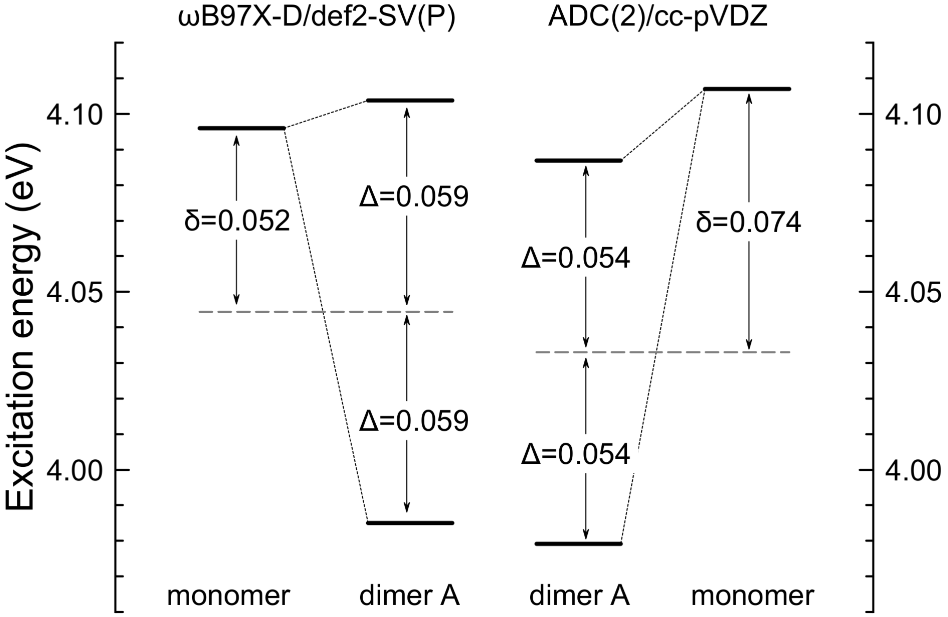

Figure S22: Energy level diagram for $\pi\pi^*$ states of the monomer and dimer A calculated with ωB97X-D/def2-SV(P) (left) and ADC(2)/cc-pVDZ (right). Exciton coupling Δ and van der Waals displacement δ are shown (in eV).

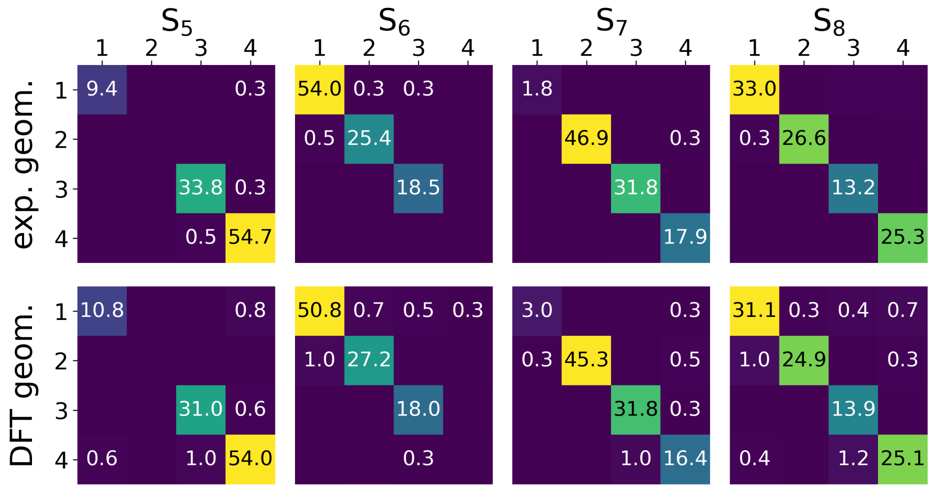

Figure S23: FTDM matrices for states S_5–S_8 of tetramer AB calculated at the experimental geometry (top row) and at the DFT-optimized (PBE+D3(BJ)) crystal geometry (bottom row).

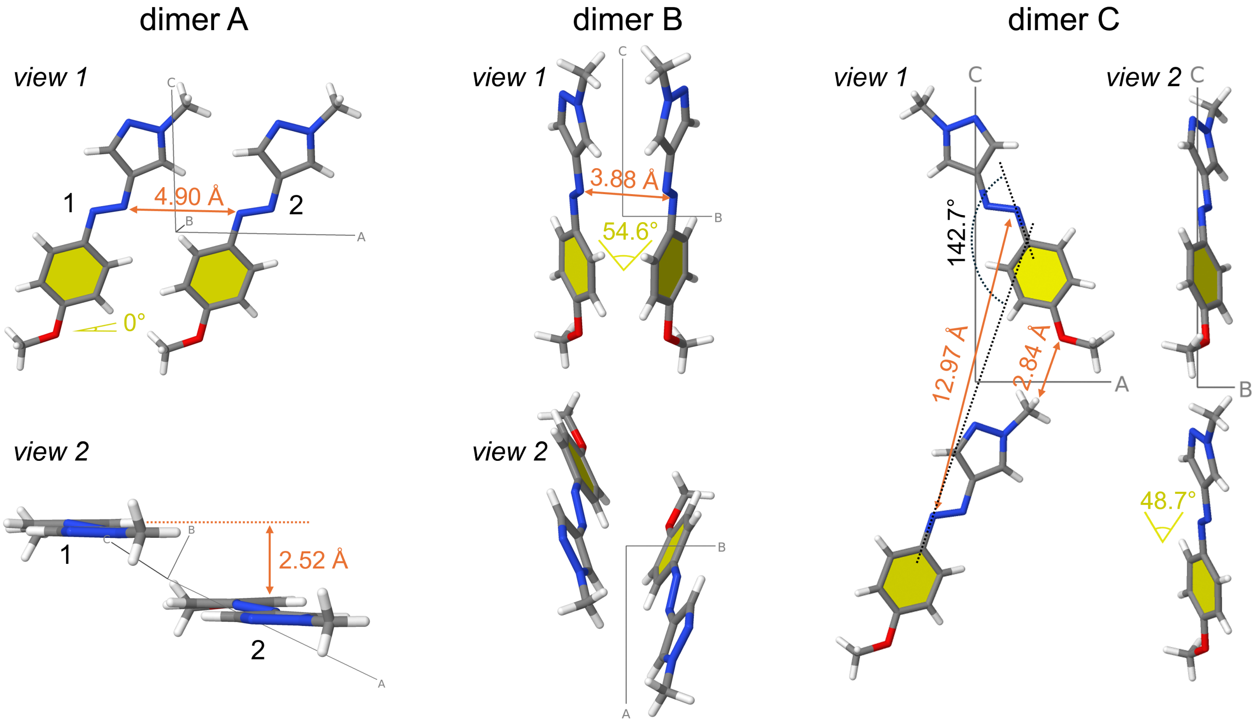

Figure S24: Different views of the dimers with key structural parameters. The phenyl rings are shaded yellowish, and the angles between their planes are shown in the same color.

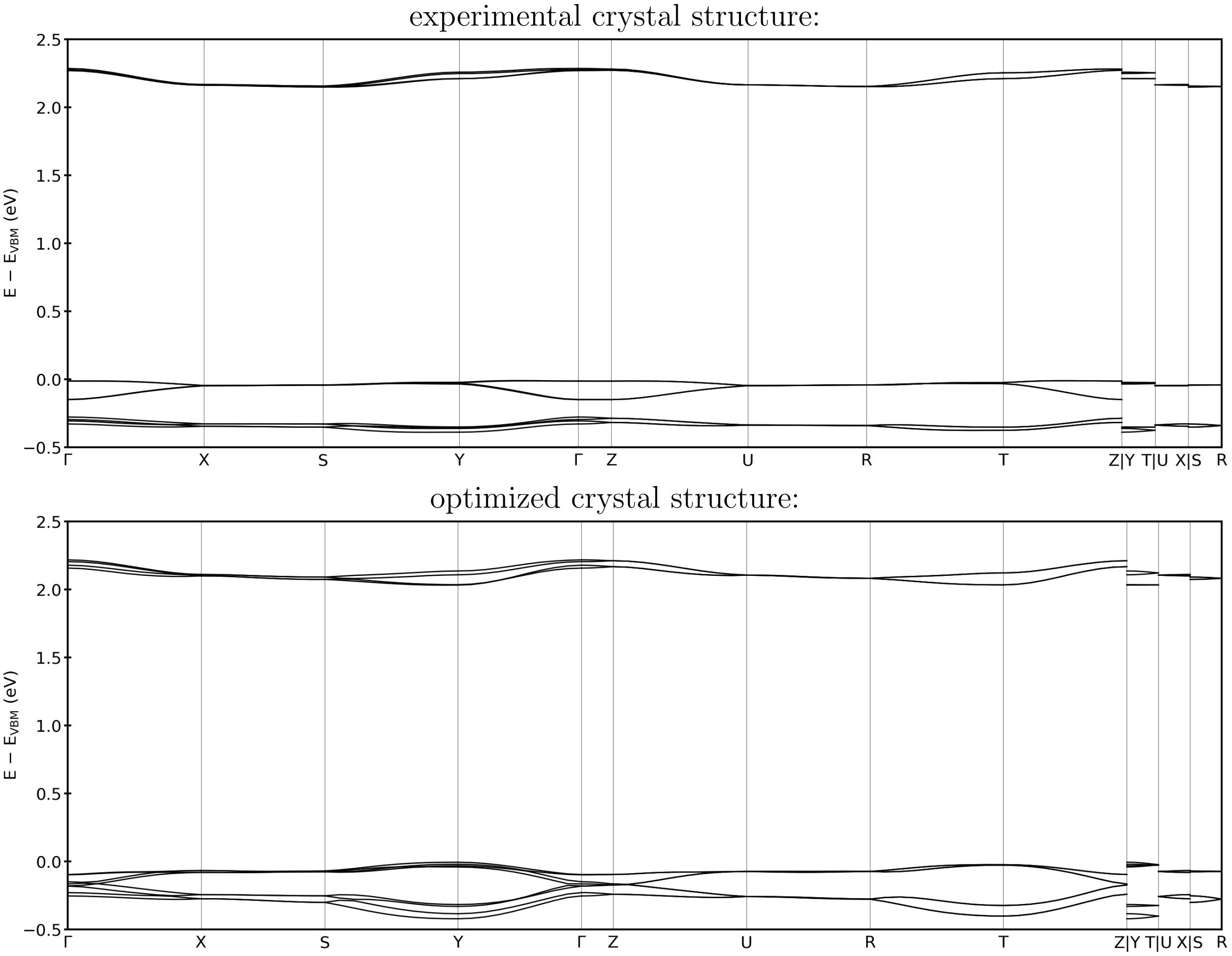

Figure S25: Band structure obtained using periodic PBE calculations on the experimental (top row) and the optimized (bottom row) crystal structure. The energy E is given relative to the valence band maximum (VBM).

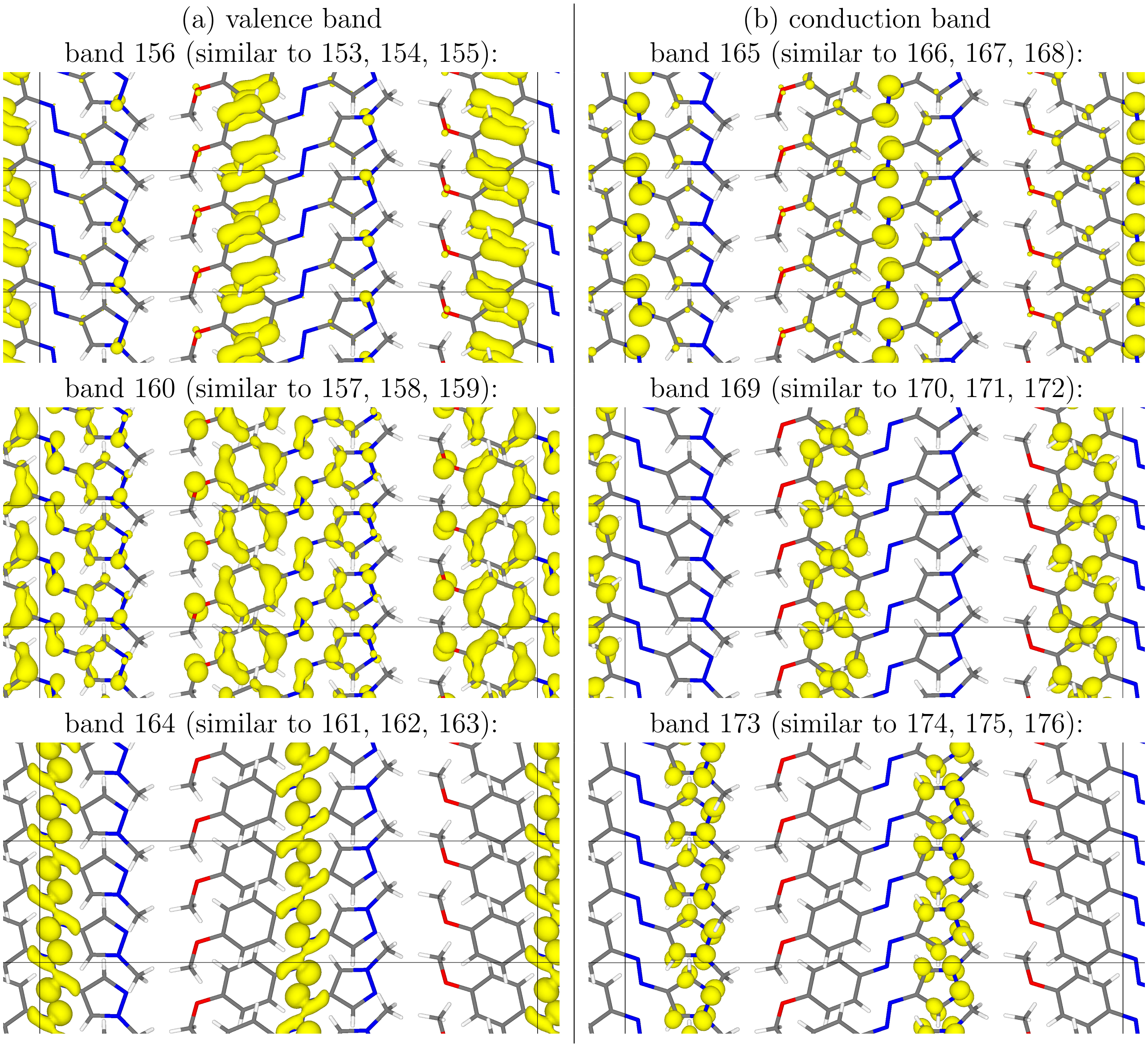

Figure S26: Partial charge density (yellow) of individual bands of the methoxy-AAP1 crystal (experimental geometry) obtained using periodic PBE calculations.
